# Prediction Errors but Not Sharpened Signals Simulate Multivoxel fMRI Patterns during Speech Perception

**DOI:** 10.1371/journal.pbio.1002577

**Published:** 2016-11-15

**Authors:** Helen Blank, Matthew H. Davis

**Affiliations:** MRC Cognition and Brain Sciences Unit, Cambridge, United Kingdom; McGill University, CANADA

## Abstract

Successful perception depends on combining sensory input with prior knowledge. However, the underlying mechanism by which these two sources of information are combined is unknown. In speech perception, as in other domains, two functionally distinct coding schemes have been proposed for how expectations influence representation of sensory evidence. Traditional models suggest that expected features of the speech input are enhanced or sharpened via interactive activation (Sharpened Signals). Conversely, Predictive Coding suggests that expected features are suppressed so that unexpected features of the speech input (Prediction Errors) are processed further. The present work is aimed at distinguishing between these two accounts of how prior knowledge influences speech perception. By combining behavioural, univariate, and multivariate fMRI measures of how sensory detail and prior expectations influence speech perception with computational modelling, we provide evidence in favour of Prediction Error computations. Increased sensory detail and informative expectations have additive behavioural and univariate neural effects because they both improve the accuracy of word report and reduce the BOLD signal in lateral temporal lobe regions. However, sensory detail and informative expectations have interacting effects on speech representations shown by multivariate fMRI in the posterior superior temporal sulcus. When prior knowledge was absent, increased sensory detail enhanced the amount of speech information measured in superior temporal multivoxel patterns, but with informative expectations, increased sensory detail reduced the amount of measured information. Computational simulations of Sharpened Signals and Prediction Errors during speech perception could both explain these behavioural and univariate fMRI observations. However, the multivariate fMRI observations were uniquely simulated by a Prediction Error and not a Sharpened Signal model. The interaction between prior expectation and sensory detail provides evidence for a Predictive Coding account of speech perception. Our work establishes methods that can be used to distinguish representations of Prediction Error and Sharpened Signals in other perceptual domains.

## Abstract 2

Neuroimaging and computational modelling explain how the human brain uses prior expectations to improve our perception of degraded speech.

## Author Summary

Perception inevitably depends on combining sensory input with prior expectations. This is particularly critical for identifying degraded input. However, the underlying neural mechanism by which expectations influence sensory processing is unclear. Predictive Coding theories suggest that the brain passes forward the unexpected part of the sensory input while expected properties are suppressed (i.e., Prediction Error). However, evidence to rule out the opposite mechanism in which the expected part of the sensory input is enhanced or sharpened (i.e., Sharpening) has been lacking. In this study, we investigate the neural mechanisms by which sensory clarity and prior knowledge influence the perception of degraded speech. A univariate measure of brain activity obtained from functional magnetic resonance imaging (fMRI) is in line with both neural mechanisms (Prediction Error and Sharpening). However, combining multivariate fMRI measures with computational simulations allows us to determine the underlying mechanism. Our key finding was an interaction between sensory input and prior expectations: for unexpected speech, increasing speech clarity increases the amount of information represented in sensory brain areas. In contrast, for speech that matches prior expectations, increasing speech clarity reduces the amount of this information. Our observations are uniquely simulated by a model of speech perception that includes Prediction Errors.

## Introduction

The observation that our perception of the world not only depends on sensory input but also on our prior knowledge has been of longstanding interest in psychology [1] and neuroscience [2–5]. There is widespread agreement that sensory input and prior knowledge are combined in neural representations; by which we mean the specific patterns of neural activity that are associated with the content of our sensory experiences. However, despite extensive experimental work in many sensory modalities [6–16], the neural and computational mechanisms by which prior knowledge guides perception are unclear [17,18].

One proposal is that neural representations of expected sensory signals are enhanced or tuned [19,20]. Critically, in this account, perceptual representations are sharpened by relevant prior expectations in much the same way as if the quality of the sensory input was increased [17,18]. Alternatively, Predictive Coding schemes suggest that expected sensory input is explained away and unexpected information is represented in the form of prediction errors (cf. in engineering [21,22] and neuroscience [3,23,24]). One intuitively attractive aspect of Predictive Coding, both for engineering and neuroscience, is its assumption that minimal effort should be invested in further processing of sensory information that is already known or expected.

Our goal in this work is to distinguish these two fundamental coding schemes for how prior expectations influence perception. Do neural representations of sensory signals contain only the unexpected parts of the sensory evidence (from now on we will refer to these as “Prediction Errors”)? Or do they contain an enhanced version of the expected sensory evidence (from now on “Sharpened Signals”)? Our approach allows us to test each of these coding schemes against behavioural and fMRI data to determine how expected sensory signals are neurally coded.

Sharpening and Predictive Coding schemes have proved hard to distinguish in neuroscience [2,5,25]. Predictive Coding theories have proposed that each level of a cortical hierarchy contains two functionally distinct subpopulations (i.e., prediction and prediction error units [3,20,24,26]). In these accounts, the signals that are passed forward from one level of the hierarchy to the next (i.e., the feedforward signals) represent Prediction Error. This Prediction Error signal is also used to update prediction units within the same level of the cortical hierarchy (through lateral interactions), such that prediction units represent a sharpened version of the sensory signal [3]. Therefore, evidence for Sharpened Signal representations has been used to support both Predictive Coding theories [20] as well as pure Sharpening theories without computation of Prediction Errors [27]. However, evidence for Prediction Error representations would be uniquely consistent with Predictive Coding and challenge pure Sharpening accounts.

Speech perception provides a biologically significant domain in which prior knowledge has been clearly shown to guide perception (for review, see [28]). Behavioural experiments show that numerous sources of proximal and distal prior knowledge (including subtitles, lip-reading, lexical constraint, or semantic predictability) can enhance subjective and objective perceptual outcomes for degraded speech [29–33]. The dominant computational theories of speech perception have included interactive-activation mechanisms that lead to enhanced representations of expected signals (i.e., Sharpened Signals), most notably in the TRACE model [34] but also in other influential models of speech perception [35–38]. More recent work has proposed Predictive Coding schemes, which use Prediction Error signals [4,7,39] to explain how prior expectations improve sensory processing. However, evidence to overturn Sharpening accounts has been lacking.

One challenge for existing research is that both suggested computational schemes predict reduced neural activity during perception of expected speech signals, either due to suppression of unexpected noise (in Sharpened Signals) or suppression of expected signals (in Prediction Errors). Brain regions in and around the left posterior superior temporal sulcus (STS) are proposed to support perceptual processing of speech [40,41] and integrate expectations from different modalities with speech input [8,39,42–46], and activity in this region is proposed to show effects of prior training on speech responses [47–49]. While these studies provide abundant evidence that prior knowledge can influence the magnitude of activity in the posterior STS during speech perception, they do not determine the computational mechanism by which relevant prior knowledge enhances perception of speech.

However, multivariate analyses of the representational content of brain responses can differentiate these two accounts by testing whether representations of speech signals are enhanced (in line with Sharpened Signals) or suppressed (Prediction Errors) when they match prior expectations. Therefore, we used representational similarity analysis [50] on multivoxel response patterns in the posterior STS. This approach is “information based” because it measures how much information about the phonetic form of speech is contained in spatial fMRI activation patterns in each of the experimental conditions that we tested [51,52]. We focus on the posterior STS because this is both a region in which effects of prior knowledge on speech processing have been repeatedly shown and also a region in which syllable identity can be decoded from multivariate BOLD signals [53–57].

**Fig 1:**
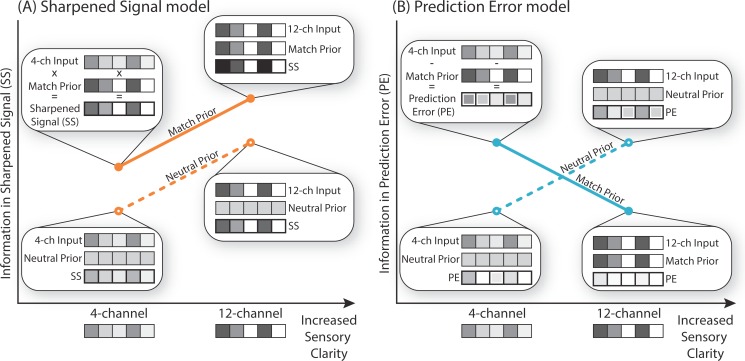
Two computational models for how matching or neutral prior expectations influence processing of sensory signals at different levels of clarity: **(A)** Sharpened Signal model and **(B)** Prediction Error model. For both accounts, neural representations are derived by combining the sensory input with prior expectation. However, the underlying computations and information content in neural representations differ. **(A)** Sharpened Signal model: Prior expectation is used to multiply sensory input, leading to more specific representations for expected compared to unexpected sensory input (Sharpened Signals, SS). This leads to additive effects of sensory detail and matching prior expectation on the information content of neural representations. **(B)** Prediction Error model: Prior expectation is subtracted from the sensory input such that neural representations encode the difference between expected and actual input (Prediction Error, PE). This leads to an interaction between sensory detail and prior expectations, with most informative neural representations found when clearer signals follow neutral expectations, or when degraded signals match informative prior expectations. Critically, when clear signals match informative prior expectations, this produces a small and uninformative Prediction Error (Match 12-channel condition). The information content of neural representations (*y*-axis) contained in SS (A) and in PE (B) refers to the signal that is passed forward after the input and prior have been combined (bottom bars). This allows us to test which of these neural representations best describes measured fMRI pattern information. In each model, neural activity patterns are represented by greyscale values over sets of units. Negative Prediction Error values are shown with a white outline.

To guide our interpretation of this data, we constructed two computational simulations based on either Sharpened Signals or Prediction Errors. Both these simulations can explain observations of perceptual enhancement and reduced fMRI responses in the left posterior STS for degraded speech that matches prior expectations. Crucially, however, these simulations make distinct predictions for the results of multivariate representational similarity analysis. In our Sharpened Signal model, simulated neural representations are enhanced for degraded speech that matches prior expectations in the same way as for speech that is presented with more sensory detail (Fig 1A). However, in our Prediction Error model (Fig 1B), these two manipulations have an interactive effect on simulated neural representations: the effect of increasing sensory detail depends on whether or not speech matches prior expectations. Increased sensory detail for expected speech leads to reduced information about the phonetic form of speech in simulated Prediction Errors. In contrast, increased sensory detail for unexpected speech leads to more Prediction Error and, hence, more information in simulated neural representations. In our experimental work, we test both these proposals using representational similarity analysis (RSA) fMRI applied to BOLD responses time-locked to the onset of a degraded spoken word.

**Fig 2:**
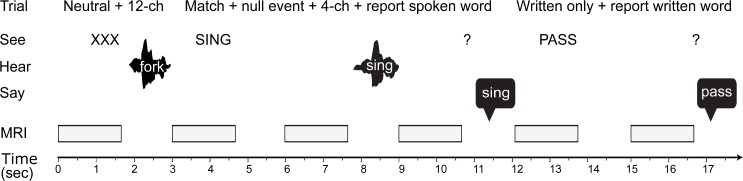
Design and experimental conditions. We used sparse imaging to record fMRI responses while participants see written words, hear subsequently presented degraded spoken words, and say what word they heard or read previously. We used two levels of sensory detail (4- and 12-channel) for presentation of the spoken words and conditions containing different pairings of written and spoken words: (1) matching written text + spoken words (“SING” + sing); (2) neutral written text (“XXXX”) + spoken words (e.g., fork); and (3) written-only text (“PASS”). Following 1/6 of all trials, participants were cued with a question mark to say aloud the previous written or spoken word. In addition, we inserted fixation crosses, null events, and trials in which written text partially or totally mismatched with spoken words (see Materials and Methods for details).

To obtain experimental evidence to differentiate these two computational accounts, we therefore simultaneously manipulated (1) prior knowledge of speech content by having participants read matching/mismatching written words or neutral text (“XXXX”) before spoken words [8,33,58] and (2) sensory detail in speech by presenting vocoded spoken words at one of two different levels of acoustic degradation (Fig 2) [59,60]. In this way, we could test whether representations of the phonetic form of speech in the posterior STS [55,57,61] are enhanced similarly by changes in prior knowledge as by changes to sensory detail (in line with Sharpened Signals) or whether these two factors interact (in line with Prediction Errors).

## Results

### Behavioural Results

**Fig 3:**
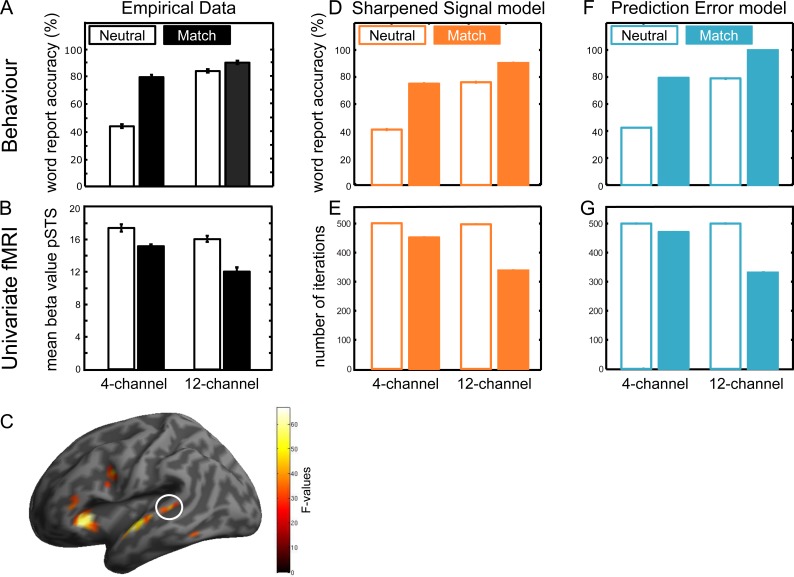
Comparison of behavioural and univariate fMRI results with model output. **(A)** Behavioural results. Matching expectations and increased sensory detail improved perception of degraded spoken words. **(B)** Univariate results. Mean beta values extracted from the posterior STS (pSTS, MNI: x = -52, y = -38, z = 6) show reduced BOLD signal during Match conditions (solid) in contrast to Neutral conditions (open). Error bars for the empirical data indicate standard error of the mean after between-subject variability has been removed, which is appropriate for repeated-measures comparisons [62]. **(C)** Main effect of prior expectations rendered on a canonical brain (*p* < 0.05 voxelwise FWE, n = 21). White circle marks the region of interest in the posterior STS. **(D/E)** Sharpened Signal model (orange) and **(F/G)** Prediction Error model (blue). For comparison with the behavioural results (**D**/**F**) we assessed word recognition accuracy in the model based on the final lexical representation (i.e., which word the model selected as presented), and for comparison with the univariate results (**E/G**) we assessed the number of activation updates required to reach the stopping criterion. Error bars for both simulations indicate the standard error of the mean over 1,000 replications. Please refer to S1 Data at https://osf.io/2ze9n/ (doi: 10.17605/OSF.IO/2ZE9N) for the numerical values underlying these figures.

First, we confirmed that, consistent with both Predictive Coding and Sharpening, providing informative prior expectations improves perception of degraded speech. Participants’ report of the degraded spoken words was improved by both increased sensory detail and matching prior information from a preceding written word (Fig 3). A two-way repeated measures ANOVA with the factors sensory detail (4- versus 12-channel) and prior knowledge (Match versus Neutral) revealed significant main effects of sensory detail on word report (12-channel: 85.39% > 4-channel: 57.83% correct; *F*(1, 20) = 133.419, *p* < 0.001, eta squared = 86.96) and prior knowledge (Match: 84.42% > Neutral: 63.49% correct; *F*(1, 20) = 89.582, *p* < 0.001, eta squared = 81.75), and a significant interaction (*F*(1, 20) = 74.997, *p* < 0.001, Fig 3A).

These effects of sensory detail and prior knowledge combined such that 4-channel vocoded speech in the Match condition was reported with equivalent accuracy as 12-channel vocoded speech in the Neutral condition (79.17% versus 83.53% correct, *t*(20) = -1.427, *p* = 0.169). Nonetheless, word report was further enhanced in the Match 12-channel condition compared to the Neutral 12-channel condition (89.68% versus 83.53% correct, *t*(20) = 3.267, *p* = 0.004) and the Match 4-channel condition (89.68% versus 79.17% correct, *t*(20) = -4.460, *p* < 0.001). Word report in the Match 12-channel condition was also more accurate than in a condition in which the spoken word was omitted and participants were prompted to report the preceding written word (89.68 versus 82.14% correct in the written only condition, *t*(20) = 2.348, *p* = 0.029). These findings confirmed that participants used prior knowledge to enhance perception of degraded speech even when relatively clear 12-channel speech was presented. Behavioural responses in the Mismatch conditions resemble the pattern of results in the Neutral condition (see S1 Fig).

### Univariate fMRI Results

Second, we sought to localise the univariate BOLD activity decrease for degraded spoken words that follow matching written words relative to words following neutral cues. These observations replicate previous findings but do not distinguish between accounts in which this effect is due to suppression of unexpected noise (Sharpened Signals) or suppression of expected signals (Prediction Errors). Univariate BOLD responses were influenced by both increased sensory detail and matching written text. A two-way repeated measures ANOVA with the factors sensory detail (4- versus 12-channel) and prior knowledge (Match versus Neutral) revealed a main effect of matching versus neutral prior knowledge on responses in the left posterior STS, as predicted, and in other regions of the speech processing network (Fig 3B and 3C, S1 Table: main effect of Match/Neutral, *p* < 0.05 FWE voxel correction). Mean beta values extracted from the left posterior STS showed a reduction during Match in contrast to Neutral conditions (Fig 3; inspection of contrast estimates from all other clusters also revealed less activity for Match than Neutral). In addition, there was a main effect of sensory detail in bilateral insula, SMA, left premotor, and orbitofrontal cortex (S2 Table; main effect of 4/12-channel, *p* < 0.05 FWE). Inspection of contrast estimates revealed less activity for 12- than 4-channel in most clusters; the reverse pattern was only observed in the right middle orbitofrontal gyrus). The interaction of prior knowledge and sensory detail did not reach corrected significance (S3 Table).

Increased BOLD activity for Mismatch > Match resembles the difference in BOLD activity found for Neutral > Match (see S1 Fig and S4 Table). This confirms that our observed effects are not due to differences in attention, anticipation of more difficult trials, or baseline differences between the Match and Neutral conditions (see S1 Text), but rather due to the influence of matching prior knowledge on speech perception.

### Model Simulation of Behavioural and Univariate fMRI Results

The behavioural and univariate results appear to be in line with both Sharpening and Predictive Coding theories. Although the underlying coding schemes differ, both accounts suggest that increased sensory detail and matching prior information should improve recognition performance and that prior matching knowledge should reduce univariate fMRI responses. To confirm this, we constructed two computational models of spoken word recognition, which only differed by using representations of Sharpened Signals or Prediction Errors to simulate how sensory information and prior knowledge are combined (see S2 Fig for details). In both these models, behavioural performance (i.e., word recognition) was simulated by the model’s ability to identify the correct word presented in degraded speech, and univariate fMRI results (i.e., the magnitude of hemodynamic activity in the left posterior STS) were simulated by the number of processing iterations required for the model to settle. By simulating the univariate fMRI signal with the number of model iterations, we assume that the hemodynamic signal as measured by fMRI integrates over several seconds of neural activity and that a longer duration of neural processing should result in an increased amplitude of the fMRI signal [63]. Six parameters were optimised for each model: the amount of sensory degradation used to simulate 4- and 12-channel vocoded speech (which influences word report and processing time), variability and confidence in behavioural responses (which influences word report), and the rate and duration of model updating (which primarily influences processing time; see S3 Fig for sensitivity analysis of the optimized parameters).

We used Akaike weights to compare goodness of fit to word report and univariate hemodynamic responses in the left posterior STS (see Materials and Methods for details). Based on 1,000 replications using the best-fitting set of parameters, a probability density function for the predicted outcome of behavioural and univariate results was generated for both model simulations. We then used the evidence ratio of Akaike weights to compare the relative likelihood of the two models given the observed data. The ratio of the Akaike weights revealed a slightly higher likelihood of Sharpened Signal model than of the Prediction Error model for both the behavioural results (w_PE/_w_Sharp_ = 0.9307) and the univariate results (w_PE/_w_Sharp_ = 0.8149). Both of these values are close to 1, indicating that there is a negligible difference between the two models [64]. The good fit observed between these models and behavioural and univariate hemodynamic data from the current experiment suggests that computation of Sharpened Signals and Prediction Errors can explain the effect of increased sensory detail and matching prior information during perception of degraded words (model simulations and experimental results shown in Fig 3).

### Multivariate fMRI Results

Although both models can accurately simulate behavioural and univariate fMRI results, they perform different underlying computations and make different assumptions about the effect of matching prior knowledge on neural representations of speech signals. The Sharpened Signal model predicts that degraded speech is better represented in the STS when it matches prior knowledge, because expected sensory features of the speech input are enhanced and unexpected sensory features are suppressed. In contrast, the Prediction Error model assumes that the expected part of the speech input is explained away (i.e., reduced) and only Prediction Errors (i.e., the difference between heard and expected speech) are represented in the STS. To test these two simulations, we assessed the neural representation of speech information by means of RSA [50]. This approach allowed us to quantify the amount of information about the phonetic form of speech that is carried by the spatial pattern of fMRI activity in each of our four critical conditions.

**Fig 4:**
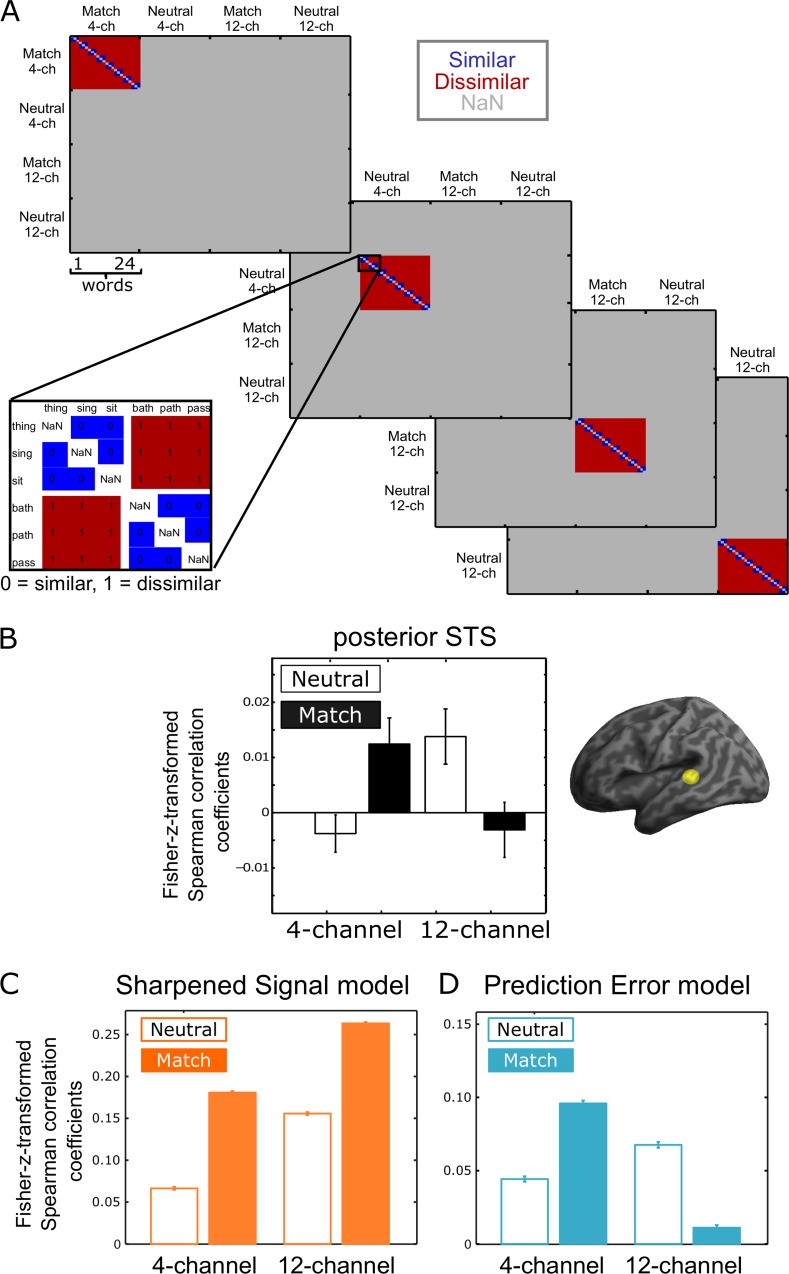
Multivariate fMRI results and simulation. **(A)** Hypothesized representational dissimilarity matrices. These four matrices were used to test similarity between words that share vowels within each of the four critical conditions (Match 4-channel, Neutral 4-channel, Match 12-channel, and Neutral 12-channel). Similarity between responses to identical items (on the main diagonal) was excluded, as was similarity between items in different conditions (“Not a Number” [NaN] values depicted in grey). Similarity between items containing the same vowel was predicted (zeroes in blue), whereas items containing different vowels were predicted to have more dissimilar representations (ones in red). These matrices are correlated with observed and simulated representational similarity. **(B)** RSA results. Fisher-z-transformed Spearman correlation coefficients for each of the four conditions in the left posterior STS (extracted from an independent ROI, [57]) show a significant interaction between sensory detail and prior expectation. Error bars indicate standard error of the mean after between-subject variability has been removed, which is appropriate for repeated-measures comparisons [62]. **(C,D)** Model comparison. Fisher-z-transformed Spearman correlation coefficients for each of the four conditions in the two models. **(C)** Sharpened Signal model (in orange) shows that both prior knowledge and sensory detail increase similarity for words that share the same vowel. **(D)** Prediction Error model (in blue) shows opposite effects of sensory detail in neutral and matching prior knowledge conditions, consistent with the RSA results (B). Error bars in (C) and (D) indicate standard error of the mean over 1,000 replications of these simulations. Please refer to S1 Data at https://osf.io/2ze9n/ (doi: 10.17605/OSF.IO/2ZE9N) for the numerical values underlying these figures.

We designed our experiment to test for categorical representations of syllable similarity, because previous studies (in fMRI [55,57] and intracranial recordings [61]) showed that categorical representations of speech, such as vowels and syllables rather than acoustic cues, are decodable from the STS. Neural representational similarity was first measured by computing a representational dissimilarity matrix (RDM) for multivoxel fMRI responses for each item and condition (see Materials and Methods for details). To quantify the amount of speech information, we computed the Fisher-z-transformed Spearman correlation between the observed RDM and a hypothesised RDM of interest that tested for increased similarity between pairs of syllables that shared the same vowel and had other segments in common (e.g., “sing” and “thing”) compared to pairs of unrelated words (e.g., “sing” and “bath”, see Fig 4A). This similarity measure was computed separately for each condition. This analysis targets speech representations in the posterior STS by testing for similarity of words that have similar phonetic forms but different lexical or semantic representations. We did not compare identical words presented in different scanning sessions.

#### Regions of interest (ROIs) analysis

Fisher-z-transformed correlation coefficients for searchlight locations were computed for two left posterior STS ROIs. The first of these was based on a 6-mm sphere centred on a coordinate defined by multivariate syllable coding in independent data in the left posterior STS (MNI: x = -57, y = -39, z = 8, [57]). The second ROI was defined by the univariate analysis of the present data (centre of mass MNI: x = -56, y = -35, z = 6). Mean correlation coefficients for these ROIs were entered into a repeated measures ANOVA with factors sensory detail (4- versus 12-channel) and prior knowledge (Match versus Neutral). This showed a significant cross-over interaction of sensory detail and prior knowledge (independent ROI: *F*(1,20) = 9.306, *p* = 0.006; and univariate ROI: *F*(1,20) = 5.449, *p* = 0.030) and no main effects of sensory detail (independent ROI: *F*(1,20) = 0.037; and univariate ROI: *F*(1,20) = 0.675) and prior knowledge (independent ROI: *F*(1,20) = 0.005; and univariate ROI: *F*(1,20) = 0.043, Fig 4B). For the Neutral condition, greater sensory detail leads to an increase in representational similarity (12- versus 4-channel speech, independent ROI: *t*(20) = 2.551, *p* = 0.0095; univariate ROI: *t*(20) = 2.542, *p* = 0.0097), whereas for the Match condition, increased sensory detail led to reduced representational similarity (comparison of 12- versus 4-channel speech, independent ROI: *t*(20) = -1.884, *p* = 0.037), though this was not significant in the univariate ROI (*t*(20) = -1.082, *p* = 0.146). Post-hoc one-sample *t* tests revealed that representational similarity was significantly greater than zero for the Match 4-channel and Neutral 12-channel conditions (independent ROI: *t*(20) = 2.263, *p* = 0.018; univariate ROI: *t*(20) = 1.792, *p* = 0.044 and independent ROI: *t*(20) = 1.913, *p* = 0.035; univariate ROI: *t*(20) = 2.179, *p* = 0.021, respectively), but not for the Match 12-channel and Neutral 4-channel conditions (independent ROI: *t*(20) = -0.559, *p* = 0.709; univariate ROI: *t*(20) = 0.018, *p* = 0.493 and independent ROI: *t*(20) = -0.880, *p* = 0.805; univariate ROI: *t*(20) = -0.725, *p* = 0.762, respectively). For completeness, we also tested other STS ROIs using clusters observed in the univariate analysis. There were no significant effects of sensory detail, prior knowledge, or interaction in either the left anterior STS (Sensory detail: *F*(1,20) = 1.96, *p* = 0.177) or the right STS (all other effects F < 1). In addition, we used the two regions in the inferior frontal gyrus (IFG) identified by the univariate analysis on prior knowledge, but Fisher-z-transformed correlation coefficients extracted from either of the regions of interest in the IFG did not reveal any significant main effect or interaction (see S1 Text, S4 Fig, and S1 Table).

To illustrate how our results depend on the assumptions made about the similarity of specific syllable pairs, we also explored other ways of testing representational similarity for speech at different levels of abstraction. We therefore compared representational similarity in the independent STS ROI to hypothesised dissimilarity on the basis of early acoustic, feature, and segmental properties (see S5 Fig). Both of the more abstract RDMs (Syllable and Segment) showed a significant interaction between prior knowledge and sensory detail (see S1 Text). This is consistent with the proposal that representations of phonetic form in the STS/superior temporal gyrus (STG) reflect the abstract, categorical similarity of syllables independent of their acoustic realisation (see S1 Text) [55,57]. The low correlation values observed in these analyses are comparable with those observed in similar studies with speech stimuli [53,57]. Analysis of cross-subject consistency of observed RDMs suggests some potential for alternative hypothesis RDMs to provide higher correlation values with the observed RDMs, but confirms the crossover interaction between sensory detail and prior knowledge (see S1 Text and S6A Fig).

#### Whole brain analysis

In order to further test for differences in representational similarity between conditions, we conducted a repeated measures ANOVA with factors sensory detail (4- versus 12-channel) and prior knowledge (Match versus Neutral) using searchlight similarity values for the whole brain. This revealed a significant interaction in the left middle occipital gyrus (*p* < 0.05, FWE voxelwise corrected) and an interaction in the left posterior STS and the left precentral gyrus at a more lenient threshold (*p* < 0.001 uncorrected, *k* > 10 voxels, S7 Fig, S5 Table). The interaction in the posterior STS showed the same pattern as the ROI analysis for the posterior STS (as depicted in Fig 4B, S5H Fig). This cluster in the left posterior STS was significant, with small volume correction (MNI: x = -57, y = -40, z = 10, *p* = 0.003) based on an independent coordinate (defined by multivariate syllable identity coding in the left posterior STS MNI: x = -57, y = -39, z = 8, [57]). Even at this lenient threshold there was no main effect of prior information on multivoxel fMRI pattern similarity and only an effect of sensory detail in the right postcentral gyrus that failed to reach corrected significance (MNI: x = 54, y = -13, z = 40, *p* < 0.001 uncorrected, k = 14).

### Model Simulation of Multivariate fMRI Results

To test our two computational simulations of spoken word recognition, we applied the same multivariate analysis to representations of the sensory input in the Sharpened Signal and Prediction Error models for each of our four conditions (for details, see Materials and Methods). As for the multivoxel fMRI RSA, we quantified the difference in pattern similarity between pairs of similar and dissimilar syllables (e.g., “sing” and “thing” versus “sing” and “bath;” see Fig 4A). The simulation for the Sharpened Signal model showed increased similarity for both increased sensory detail and matching prior information (Fig 4C). In contrast, the simulation for the Prediction Error model showed an interaction between sensory detail and prior information (Fig 4D). Specifically, there was greater pattern similarity for similar syllable pairs in the Neutral 12-channel than in the Neutral 4-channel condition, whereas in the Match 12-channel there was less pattern similarity than in the Match 4-channel condition. This outcome resembles the interaction of sensory detail and prior knowledge shown for multivariate fMRI results in the posterior STS ROI (Fig 4B). In addition, we repeated the cross-subject consistency analysis on representations generated by individual simulated participants. For the Prediction Error but not for the Sharpened Signal model, this showed the same crossover interaction of sensory detail and prior knowledge as in the equivalent fMRI analysis, suggesting a common underlying explanation (see S1 Text and S6A–S6C Fig).

Again, we used the evidence ratio of Akaike weights to compare the evidence for both models given the pattern similarity results in the left posterior STS (see Materials and Methods). Importantly, both models used parameters optimised to simulate the behavioural and univariate fMRI results, and no modifications or parameter optimisation were performed when simulating similarity in spatial patterns of fMRI activity. For the multivariate fMRI results, the evidence ratio of the Akaike weights revealed that the multivariate fMRI patterns very strongly supported the Prediction Error model over the Sharpened Signal model (w_PE/_w_Sharp_ = 1.898 x 10^11^, tested based on the independent ROI in the posterior STS [57]). Hence, computational simulations provided compelling evidence that multivariate fMRI results are more consistent with computation of Prediction Errors than of Sharpened Signals in the posterior STS during the perception of degraded speech.

## Discussion

We used multiple approaches (behavioural, computational, univariate, and multivariate fMRI) to investigate how prior expectations improve perception of degraded speech in order to distinguish Sharpened Signal and Prediction Error computations. Our experimental findings, first of all, replicate the existing literature [31–33,65] by showing that behavioural report of degraded words was improved both by matching expectations and by increased sensory detail (Fig 3A). Second, we show that matching expectations reduced BOLD activity during speech processing in left posterior STS (Fig 3B and 3C). Like other previous observations in the literature [8,39,43,45,46,66], these findings are in line with either Sharpened Signal or Prediction Error computations for combining prior knowledge and sensory input. This is confirmed by our computational simulations, which show that a good fit to behavioural and univariate fMRI data is achieved by models that include either of these two coding schemes (Fig 3D–3G). These model simulations are also consistent with the proposal that BOLD responses in the Match condition are lower because word identification is easier (as suggested by the behavioural improvements we observed). More informative results come from fMRI multivoxel pattern similarity, which revealed an interaction between prior knowledge and sensory detail in the posterior STS (Fig 4B). Specifically, for degraded speech that follows neutral expectations, increased sensory detail improved the amount of sensory information contained in fMRI multivoxel patterns. However, for speech that matched expectations, increased sensory detail led to a reduction in the amount of information represented in the posterior STS as measured by similarity analysis. This interaction is uniquely consistent with a Prediction Error model in which expected sensory input is explained away, and deviations from expectation are represented as Prediction Errors (Fig 1B). Our results, therefore, provide evidence for computation of Prediction Errors but not of Sharpened Signals (see simulations in Fig 4C and 4D).

Why is this interaction between sensory detail and prior knowledge shown in multivariate representations of speech so diagnostic of Prediction Error computations? In explaining this interaction, we will first consider the situation in which listeners have uninformative prior expectations. In the absence of specific expectations (as in the Neutral condition), both Sharpening and Prediction Error accounts propose that the amount of sensory information represented in neural patterns should increase with the amount of sensory detail in the input. In Prediction Error schemes, the brain does not pass forward the sensory input directly, but rather the discrepancy between expectations and sensory input. These Prediction Errors will provide an informative representation of the sensory input if these expectations are uninformative and the sensory input is sufficiently clear. Thus, our observation of enhanced coding for Neutral 12-channel compared to Neutral 4-channel stimuli is equally consistent with Prediction Error as with the traditional view that the brain directly represents the sensory input. The true test of Prediction Error schemes is provided by conditions in which specific and accurate expectations guide perceptual processing.

The hallmark of Prediction Error in our data is that for speech that matches prior expectations increasing the sensory detail reduces the informativeness of multivariate representations (Fig 1B). This is a counterintuitive finding, because clear speech that matches a previously presented written word (our Match 12-channel condition) is most accurately perceived, whereas multivariate representations are more informative in the less intelligible Match 4-channel condition. This is to be expected because Prediction Errors will be substantially reduced for conditions in which sensory input matches prior knowledge. Hence, increases in sensory detail lead to a better correspondence between sensory input and listeners’ prior expectations of clear speech. Our observation of reduced representation of speech content for Match 12-channel compared to Match 4-channel stimuli is entirely consistent with Prediction Errors but stands in marked contrast to the outcome expected for Sharpened Signals—or, indeed, any account in which sensory representations directly encode perceptual outcomes. Low pattern similarity for the condition with the clearest perceptual outcome (Match 12-channel) might appear surprising given previous findings that perceptual representations can be decoded from low-level response patterns [67–69]. However, these findings can be reconciled with Prediction Error schemes by recalling that these previous experiments used presentation conditions similar to the Neutral condition in our experiment (i.e., an uninformative prior).

Prediction Error can also explain the apparent increase in the informativeness of speech representations in the Match 4-channel condition compared to the Neutral 4-channel condition. Our simulations reveal that when sensory signals are severely degraded (such as for 4-channel vocoded speech), informative Prediction Errors are derived from the residual of matching expectations (in the Match condition). A specific expectation, as provided by our written word cue, when combined with a less informative stimulus, remains “unfulfilled” and is therefore represented as a negative but informative Prediction Error. Informative Prediction Errors (either positive or negative) are absent when prior expectations are uninformative (in the Neutral condition). Hence, both Prediction Error and Sharpened Signal models can explain our observation of increased representation of 4-channel speech that matches prior expectations. Other similar studies in the literature have explored whether visual representations of expected stimuli are sharpened or reduced [20,26] but have yielded contradictory findings. While Prediction Error was supported by univariate hemodynamic responses to unexpected classes of visual stimuli (faces versus houses, [26]), multivariate responses supported sharpening of expected visual gratings [20]. Two other differences between our work and this previous multivariate study are noteworthy. First, we separated the neural response to the cue (written word) and stimulus (spoken word). Second, we tested the interaction between sensory information and prior knowledge. Only Prediction Error can explain the full interaction of sensory detail and prior knowledge described above.

By the Prediction Error scheme, there should be a negative correlation between neural representations in the Neutral 12-channel condition (a positive Prediction Error) and the Match 4-channel condition (a negative Prediction Error, apparent in positive and negative Prediction Error in Fig 1B). However, an additional analysis of the present data showed that there was neither a negative nor a positive correlation between these conditions (we tested for a positive correlation because a negative Prediction Error could evoke a positive hemodynamic response due to metabolic costs of neural inhibition). These null findings cannot rule out the possibility that both conditions do indeed contain complementary information based on positive and negative Prediction Errors. Direct neural data (e.g., from intracranial recordings) might provide a more sensitive test of this proposal. Taken to an extreme (i.e., without any sensory input), computation of negative Prediction Errors could also explain previous results showing that the omission of an expected stimulus causes an increased signal [70–72] from which stimulus identity can be decoded [72–74].

### Implications for Predictive Coding Theories

Current Predictive Coding theories suggest that cortical regions involved in sensory processing contain two subpopulations of neurons: (1) prediction error units that represent the unexpected part of the incoming sensory information and (2) prediction units that represent the expected part of the incoming sensory information (and can be sharpened by matching prior expectations) [3,24,75]. These models have thus drawn support from empirical evidence showing either Prediction Errors [26,39,49,76] or Sharpened Signals [20] by attributing neural responses to prediction error and prediction units, respectively. Our goal in this study was to test two functionally distinct coding schemes in isolation by building computational models in which a simulated cortical area passes only one type of information forward (only Prediction Errors or Sharpened Signals). In the context of these simulations, our results provide clear evidence for representations of Prediction Errors. However, our multivariate fMRI findings do not oppose theories of Predictive Coding that propose Sharpened Signals coded by prediction units in addition to Prediction Errors in prediction error units [3,23,24]. The absence of evidence for Sharpened Signals in our data from the STS could be explained by previous proposals that fMRI measurements are dominated by responses from prediction error units (as [26,77] have argued for visual cortex). It could be that other neural measures, such as neurophysiological recordings with depth electrodes [78] or laminar-specific ultra-high field strength fMRI [79,80] are better able to detect responses from prediction units and could provide evidence of laminar-specific representations of Prediction Errors and Sharpened Signals.

Nonetheless, the interaction observed in the present study favours Predictive Coding theories (with representations of Prediction Error) over the traditional view that the brain directly passes forward the sensory input, as hypothesised in a Sharpening scheme without representations of Prediction Error. Our simulations show that in Sharpening schemes, the Match 12-channel condition should contain the clearest representation of speech content. This was not observed in the present data (compare Fig 4B and 4C). Our work not only provides evidence to support the hypothesis that integration of prior expectation and perceptual input for speech is achieved through computation of Prediction Errors or Sharpened Signals, but also introduces a new and critical diagnostic finding for Prediction Error responses: For unexpected stimuli, increased sensory detail should improve the amount of sensory information contained in neural patterns. However, for stimuli that match expectations, increased sensory detail should lead to a reduction in the amount of information represented. Future studies in other sensory modalities and domains might benefit from adopting similar methods.

### Implications for the Perception of Speech and Other Sensory Signals

Our work joins a number of recent fMRI and MEG/EEG studies in proposing an important role for Prediction Error computations in speech perception [4,7,8,39,81]. In these earlier studies, the observation of decreased activation for expected stimuli in the STG has been interpreted as a neural correlate of reduced Prediction Error and, hence, as evidence for Predictive Coding theories. However, almost all established computational theories of speech perception can also explain this observation. For example, TRACE [34] implements a form of neural sharpening in which prior knowledge enhances the representation of expected sensory signals and suppresses sensory noise, producing a reduced neural response overall. Similar, interactive activation models [35–38] might predict exactly the same decrease in STG activity for expected stimuli, as observed in these previous neuroimaging studies. Thus, existing empirical evidence proposed for Predictive Coding is also largely consistent with Sharpening theories. Even our previous comparison of Predictive Coding and Lexical Competition accounts of spoken word recognition [39] challenged the competitive lexical selection mechanism implemented in TRACE, but did not test the Sharpening mechanism traditionally described as Interactive Activation.

In this context, then, the results of our study have important implications for understanding speech perception, a domain in which the presence and function of top-down processes has been much debated [82,83]. By directly quantifying the information represented in multivariate signals during perception of correctly expected and unexpected speech, we provided evidence that the neural mechanisms underlying speech perception are in line with Prediction Error simulations. Prior knowledge of speech content is used to explain away sensory evidence such that speech representations encode Prediction Error.

The present multivariate interaction of sensory detail and prior information supports a Predictive Coding theory for how matching expectations improve perception of degraded speech. In contrast, enhanced representation of attended compared to unattended speech supports Sharpening mechanisms [84–87]. These findings could be reconciled by theories proposing that expectation (Prediction Error) and attention (Sharpening) operate in parallel, as suggested in some Predictive Coding theories [3,88]. However, more detailed computational specification of attentional mechanisms will be required to test these theories with experimental data. Comparing neural representations of attended and unattended speech signals at varying levels of expectation and degradation may be informative.

There are three reasons why our results are of general interest for the study of speech and other domains of perception. One key aspect of our approach is that we assessed the perception of speech presented at varying levels of signal degradation. As in accounts proposing Bayesian perceptual inference [89], this provides the best opportunity to observe influences of prior knowledge on perception. In doing so, we also test the perception of speech in listening conditions similar to the way that speech is most often heard in the real world [90]. A second form of generality is that prior expectations for speech were derived from written text. Our results may therefore also inform other situations in which prior knowledge and sensory information are combined across different modalities for speech [91–93] and other cross-modal stimuli [94–96]. Third and perhaps most important, however, is that the representations of Prediction Error that we have observed during speech perception might apply to many other sensory domains in which prior knowledge has been shown to influence perception (such as audition [6,7,76,97], vision [9–12,20,98,99], touch [13], gustation [14,100], olfaction [15], and pain [16]). The interactive effect of prior knowledge and sensory input on neural representation of degraded stimuli provides a stronger test of Predictive Coding theories of perception than has been provided by existing methods, as it offers the potential to challenge alternative views based purely on Sharpening mechanisms.

### Conclusions

In summary, the present results show that both increased sensory detail and matching prior expectations improved accuracy of word report for degraded speech but had opposite effects on speech coding in the posterior STS. Following neutral text, increased sensory detail enhanced the amount of speech information, whereas matching prior expectations reduced the amount of measured information during presentation of clearer speech. These findings support the view that the brain reduces the expected and, therefore, redundant part of the sensory input during perception, in line with representations of Prediction Error proposed in Predictive Coding theories.

## Materials and Methods

### Ethics Statement

Ethical approval was provided by Cambridge Psychology Research Ethics committee (CPREC) under approval number 2009.46. All participants provided their written informed consent.

### Participants

Twenty-five healthy native-English speakers (aged 18–40, with self-reported normal hearing and language function) participated in the experiment. Three participants had to be excluded because they were insufficiently attentive to the written text during the scanning runs (they reported less than 50% of the written words correctly when prompted). One additional participant had to be excluded due to technical problems. The reported analyses are therefore based on 21 participants (mean age 25 y [range 19 to 38 y], 9 females).

### Stimuli

Word stimuli consisted of 24 different monosyllabic words, each with a consonant-vowel-consonant structure. The words were selected as eight triples of three similar words, each sharing the same vowel and with offset and onset changes between items (eight triples: thing/sing/sit, bath/path/pass, deep/peep/peak, pork/fork/fort, doom/tomb/tooth, take/shake/shape, kite/tight/type, zone/moan/mode). These stimuli were recorded by a male native speaker of Southern British English and noise-vocoded (4- and 12-channel) using custom scripts written in Matlab [59]. The syllables were filtered into 4 or 12 approximately logarithmically spaced frequency bands from 70 to 5,000 Hz [101], with each pass band 3 dB down with a 16 dB/octave roll off. In each band, envelopes were extracted using half wave rectification, and pitch synchronous oscillations above 30 Hz were removed with a second-order Butterworth filter. The resulting envelopes were multiplied with a broadband noise and then band pass filtered in the same frequency ranges as the source and recombined. To ensure that acoustic intensity was matched across all stimuli, the RMS amplitude of each sound file was equalised. Finally, we applied an additional filter to ensure a flat frequency response when the spoken words were presented via Sensimetrics insert headphones in the scanner (http://www.sens.com).

### fMRI Procedure

Participants read written words and listened to subsequently presented degraded spoken words (see Fig 2). There were four conditions containing different pairings of written and spoken words: (1) matching written text + spoken words (“SING” + *sing*); (2) neutral written text (“XXXX”) + spoken words (*sing*); (3) partially mismatching written text + spoken words (“SIT” + *sing*); (4) totally mismatching written text + spoken words (“SING” + *doom*). In addition, we included a fifth condition in which only written text (“SING”) was presented to test whether participants attended to the written words. Only the match and neutral conditions (condition 1 and 2) were repeated sufficiently (six presentations per item per condition) to permit multivariate RSA (see below for details). In occasional catch trials, a response cue, which consisted of a visual display of a question mark, was presented 1,000 ms after trial onset. This cued participants to say aloud the written or spoken word that they saw or heard previously. This design does not allow the analysis of response times, because participants were cued to respond after a delay. A previous behavioural study in our lab showed that response times for reporting vocoded spoken words are uninformative even when collected in such a way as to permit response time analyses [102]. The partial and total mismatch conditions (condition 3 and 4) were included to make sure that participants paid attention to both the written and the spoken word; these conditions ensured that they could not simply report the preceding written word. Due to the small number of trials, RSA analysis was not possible for neural responses measured in the Mismatch condition. We can, however, report behavioural and univariate fMRI results for the Mismatch condition; this confirms that behavioural and neural enhancement following matching written text is not due to prestimulus attention or anticipation (because prestimulus processes will be identical following mismatching text but enhanced perception is not typically observed) [8,33].

Trials commenced with presentation of a fixation cross (1,000 ms), followed by presentation of a written word (500 ms), again followed by a fixation cross (500 ms), and finally the presentation of a spoken word. Written cues (i.e., written words, neutral “XXXX”, and fixation cross) were presented in grey in the centre of the black screen. Trials were 3 to 9 s long, depending on the number of inserted null events to decorrelate the events within each run (76 trials of 3 s without null event, 45 trials of 6 s with a null event of 3 s, and 15 trials of 9 s with a null event of 6 sec, resulting in 211 TRs per run with null events).

Spoken words were presented after 4- or 12-channel noise-vocoding to produce two different levels of sensory detail in the speech input. Altogether, this resulted in 816 trials, including 1/6 catch trials (136 trials) in which participants had to give their verbal response (24 neutral and 24 match words x 6 repetitions x 2 levels of sensory detail = 576 trials, 24 written-only words x 6 repetitions = 144 trials, 24 partial mismatch and 24 total mismatch words x 2 levels of sensory detail without repetition on the word level = 96 trials; i.e., 11.8% of the trials contained mismatching information). These trials were split into 6 runs of 136 trials each, ensuring that each word in each condition occurred once in each scanning run. With additional catch trials, each run took 11.7 min, and the overall experiment lasted approximately 70 min for all 6 runs. Stimulus delivery was controlled and behavioural responses were recorded with E-Prime 2.0 software (Psychology Software Tools, Inc.).

### Scanning Parameters

#### Structural scanning

MRI data were acquired on a 3-Tesla Siemens Tim Trio scanner using a 32-channel head coil. A T1-weighted structural scan was acquired for each subject using a three-dimensional MPRAGE sequence (TR 2,250 ms, TE: 2.99 ms, flip angle: 98 deg, field of view: 256 x 240 x 160 mm, matrix size: 256 x 240 x 160 mm, spatial resolution, 1 x 1 x 1 mm).

#### Functional scanning

The fMRI session was split into 6 runs of 11.7 min. We used sparse imaging to acquire fMRI data. For each participant and scanning run, 239 echo planar imaging (EPI) volumes comprising 26 slices of 3 mm thickness were acquired using a continuous, descending acquisition sequence (TR 3,000 ms, TA 1,600 ms, TE 30 ms, FA 78 deg, matrix size: 64 x 64, in plane resolution: 3 x 3 mm, inter-slice gap 25%). We used transverse-oblique acquisition, with slices angled away from the eyes to avoid artefacts from eye movements. Visual stimuli were projected on a screen at the head-end of the scanner table and reflected onto a mirror attached to the head coil above the participants’ eyes. We used Sensimetrics headphones (Sensimetrics Corporation, Malden, MA, USA, model S14) to deliver the sound stimulation and a MR-compatible microphone (FOMRI II, Optoacoustics) to record verbal response.

### Behavioural Analysis

Verbal responses recorded in the scanner were transcribed by two independent raters (the first author and a native English speaker with a PhD in phonetics who was naïve to the stimulus set) and disagreements adjudicated by a third rater (the senior author). All raters were blind to which word and stimulus condition was presented in each trial. Responses were scored for whole-word accuracy and analysed using Matlab. Because the percent correct performance scores were bound to [0;1], we applied an arcsine transformation [103] before we computed a two-way repeated measures ANOVA and the corresponding post-hoc pared *t* tests.

### Univariate fMRI Analysis

Data were analysed using SPM8 (http://www.fil.ion.ucl.ac.uk/spm) applying automatic analysis (aa) pipelines [104]. The first three volumes of each run were removed to allow for T1 equilibrium effects. Scans were realigned to the first EPI image. The structural image was coregistered to the mean functional image and the parameters from the segmentation of the structural image were used to normalise the functional images, which were resampled to 2 mm isotropic voxels. The realigned normalised images were then smoothed with a Gaussian kernel of 8 mm full width half maximum. Data were analysed using the general linear model with a 128 s high pass filter. We included the onset of 11 event types in the GLM, each convolved with the canonical SPM haemodynamic response: eight conditions come from specifying the onset of spoken words paired with four types of written text (matching, neutral, partially mismatching, and totally mismatching) crossed with two types of vocoding (4- and 12-channel). We also specified onsets for written words and neutral strings (“XXXX”) as well as the onset of the visual task cue that instructed participants to say the spoken word. Following parameter estimation of the first level model, we conducted a repeated measures ANOVA with two factors: prior knowledge (matching versus neutral text) and level of sensory detail (4- versus 12-channel) to assess the main effects and interaction of these factors.

We were interested in the effect of hearing speech that matches prior expectations on BOLD responses in the left posterior STS. To locate these ROIs for the multivoxel RSA (see below), we tested for a main effect of prior knowledge (F-contrast “Neutral versus Match”) and identified a cluster at *p* < 0.05 FWE voxel-corrected in the left posterior STS.

### Multivariate RSA fMRI Analysis

Multivariate analyses were conducted on realigned data within each participant’s native space without normalisation or spatial smoothing. An additional first-level model was constructed for each participant that contained the same set of regressors as the first level model used for the univariate analysis, except that regressors for individual spoken words were used in each of the four conditions for which there were sufficient numbers of repetitions for item-specific modelling (4- and 12-channel vocoded words following neutral or matching text). This resulted in 103 conditions per participant per run: 24 words for each of these four conditions and the remaining seven conditions from the univariate model. For each of the 96 item-specific regressors in these four conditions, we estimated single-subject T-statistic images for the contrast of speech onset compared to the unmodelled resting period, averaged over the six scanning runs.

We used the resulting single condition and item T-images for RSA [50] using the RSA toolbox [52]. We used T-images so that effect sizes were weighted by their error variance, which reduces the influence of large but variable response estimates for multivariate analyses [105]. RSA involves testing whether the observed similarity of brain responses in specific conditions (a neural RDM) corresponds to a hypothetical pattern of similarity between these conditions (hypothesis RDM). We constructed four hypothesis RDMs to test for greater similarity between syllable pairs within the same stimulus triple (i.e., syllables that shared the same vowel and had similar onset or offset segments like “sing” and “thing,” as compared to dissimilar syllables like “sing” and “bath”) within each of four critical conditions: Match 4-channel, Neutral 4-channel, Match 12-channel, and Neutral 12-channel. The design of our experiment was motivated by previous work that showed that STS encodes vowel and syllable similarity [55,61], rather than spectrotemporal acoustic cues [61]. The comparisons used in our ROI analysis test for global similarity in representations of the phonetic form of similar-sounding spoken words because multiple consonantal features as well as the vowel are preserved within each syllable triple (e.g., bath/path/pass). We chose to analyse similarity of neural representations for phonetically similar but non-identical words for two reasons: (1) this approach allowed us to merge all six runs into a single analysis, which reduced the noise in the estimation of the T-images relative to a split-half method, and (2) comparing similar but non-identical word pairs makes our method insensitive to other forms of lexical or semantic similarity that could lead to similar neural representations for identical word pairs (e.g., in regions that code for word meaning [106]). Similarity between items in different conditions and between identical items (i.e., the main diagonal) was therefore not included in our hypothesis RDMs (see Fig 4A).

We measured multivoxel RDMs by computing the dissimilarity (1–Pearson correlation across voxels) of T-statistics for a specific item and condition. In a searchlight analysis, the sets of voxels were extracted by specifying grey-matter voxels (voxels with a value > 0.33 in a probabilistic grey-matter map) within an 8-mm radius sphere of each grey matter voxel (with a voxel size of 3 x 3 x 3.75 mm, i.e., a maximum of 65 voxels per sphere). This was repeated for all searchlight locations in the brain. The similarity between the observed RDM and each of the hypothetical RDMs was computed using a Spearman correlation for each searchlight location, and the resulting correlation coefficient returned to the voxel at the centre of the searchlight. This resulted in a Spearman correlation map for each participant in each grey matter voxel. To assess searchlight similarity values across participants at the second level, the Spearman correlation maps for each participant were Fisher-z-transformed to conform to Gaussian assumptions, normalized to MNI space, and spatially smoothed with a 10-mm FWHM Gaussian kernel for group analysis. These second-level analyses used a within-subject analysis of variance similar to those used for the univariate fMRI analysis.

#### Region of interest (ROI) analysis

In a region of interest (ROI) analysis using MarsBaR (http://marsbar.sourceforge.net/), we extracted similarity values from searchlights within ROIs defined on the basis (1) of an independent coordinate (defined by multivariate syllable identity coding in the left posterior STS MNI: x = -57, y = -39, z = 8, [57]) and (2) of the univariate fMRI analysis. We used the independent ROI in the left posterior STS to make sure that the results were not caused by any potential dependencies of univariate and multivariate analyses. In addition, the univariate ROIs allowed us to test for differences in observed multivoxel similarity in each of the four conditions within STS regions defined on the basis of showing hemodynamic response reductions for degraded words following matching written words. To locate this region, we tested for a main effect of prior knowledge (F-contrast “Neutral versus Match”) and identified a cluster at *p* < 0.05 FWE voxel-corrected in the left posterior STS (centre of mass MNI: x = -56, y = -35, z = 6, k = 99 voxels). For completeness, we also considered two other STS clusters from this univariate analysis: left anterior STS (centre of mass MNI: x = -57, y = -10, z = -5, k = 229 voxels) and right STS (centre of mass MNI: x = 56, y = -13, z = -4, k = 92 voxels). For each ROI, we obtained one Fisher-z-transformed Spearman correlation value for each of our four conditions. We then tested for differences between these conditions in a repeated measures ANOVA with factors sensory detail (4- versus 12-channel) and prior knowledge (Neutral versus Match). We conducted post-hoc one-sided paired *t* tests on the data extracted from the independent ROI in the left posterior STS (MNI: x = -57, y = -39, z = 8, [57], sphere 6 mm, 896 mm volume) and based on the ROI defined by the univariate analysis (centre of mass MNI: x = -56, y = -35, z = 6, k = 99 voxels, 782 mm volume) to test for the Neutral condition whether sensory detail led to an increase in representational similarity and for the Match condition whether sensory detail led to a decrease in representational similarity. In addition, we conducted post-hoc one-sample *t* tests on the data extracted from the independent ROI in the left posterior STS [57] and the ROI defined by the univariate analysis to test whether the correlation was significantly greater than zero for the four conditions, individually.

### Computational Simulations of Spoken Word Recognition using Sharpened Signals or Prediction Errors

We used two computational implementations of Sharpened Signal and Prediction Error models of spoken word recognition (using update mechanisms based on [75]), to simulate observed behavioural performance (i.e., word recognition), univariate fMRI results (the magnitude of hemodynamic activity in the STS), and RSA fMRI results (the similarity of representations for word pairs in the left posterior STS) in each of our four experimental conditions. The sensory representations supplied at the input, the output lexical representations, and the specification of matching or neutral prior knowledge was identical for both simulations. We used a localist lexical representation (i.e., a set of 24 units, each of which was activated to represent a single word), as in previous models of spoken word recognition such as TRACE [34] or Shortlist [107]. The input to the model was provided as a distributed set of phonetic features (derived from [108]). These are similar to the acoustic/phonetic features supplied as the input to TRACE or in recurrent network simulations such as the Distributed Cohort Model [109]. However, to avoid the complexity of representing temporal information (and given the slow haemodynamic responses measured by fMRI), we assumed that speech information is provided in parallel over three groups of units for the initial consonant, medial vowel, and final consonant of our CVC words.

The key difference between the Sharpened Signal and Prediction Error models concerns the computations by which prior knowledge is combined with degraded sensory representations of expected spoken words. In the Sharpened Signal simulation, expected sensory features receive additional activation through increased sensory gain [19,20], whereas in the Prediction Error model, prior expectations contribute to perception by subtracting expected input from sensory representations (i.e., computation of Prediction Error [3,23,24]). In both simulations, an iterative settling procedure was used such that feature representations of the input are combined with prior knowledge to generate feature representations that convey Sharpened Signals or Prediction Errors respectively (hereafter “sharpened features” and “prediction error features”). These representations were used to update lexical activations, and updated lexical activations in turn led to modified top-down expectations. This settling procedure continued until a settling criterion was reached or a maximum number of iterations had been performed.

#### Representations of speech input, lexical knowledge, and perceptual expectations

The representations of the speech input, lexical knowledge, and perceptual expectations were the same for both the Sharpened Signal and the Prediction Error model (see S2 Fig). The sensory input for each degraded spoken word was determined by a feature matrix that transformed a phonological transcription of each of the 24 words into an articulatory feature representation based on phonetic descriptions of each segment [108]. We used articulatory representations because they appropriately model the similarity of different spoken words, and there is considerable evidence from intracranial recordings [110] and multivariate fMRI to support the presence of articulatory representations in superior temporal regions [106,111]. Representing the segments of the 24 words in our stimulus set required 13 consonantal features and 11 vowel features, concatenated into a set of 37 binary features for the CVC syllables used in the experiment. The 13 consonantal features were divided into four groups: (1) place of articulation (six features: bilabial, labiodental, dental, alveolar, palato-alveolar, velar), (2) manner of articulation (three features: stop, sibilant, non-sibilant), (3) nasality (three features: nasal, oral), and (4) voicing (two features: voiceless, voiced). The 11 vowel features were divided into four groups: (1) height (five features: high, mid-high, mid, mid-low, low), (2) backness (two features: front, back), (3) rounding (two features: rounded, unrounded), and (4) length/diphthong (two features: long, short). Based on these position-specific features, we constructed a feature-to-word transformation matrix that included positive binary values in each row to indicate which phonetic features were relevant for each word (see S2 Fig). Each row contained 12 active features (four features for each consonant and vowel). This matrix served as a set of connection weights to link phonetic features to words in both models and thereby encoded long-term knowledge of the form of each spoken word.

To generate different levels of degradation of the sensory input (equivalent to 4- or 12-channel vocoded speech), we set noise parameters (for low- and high-sensory detail) that determined the degree to which the appropriate input features remained active and inappropriate features inactive following degradation. Noise was added to each group of features (place, manner, etc.) individually, such that the sum of all active features within each group remained 1 and, hence, the pattern of activation within each of the feature groups could be interpreted as a probability distribution. For example, if the current “place” feature was 1 for bilabial (as in the initial segment of “bath”), this group of features would be [1 0 0 0 0] for clear speech, but with a noise parameter of 0.5 the input representation would be set to [0.5 0 0 0 0] and a uniform random amount (that sums to 0.5) assigned to all five features. Thus, with a noise parameter of 1, no information would remain concerning the place of articulation of the speech input. The noise parameter for low and high sensory detail conditions was fitted separately for each model based on the aggregate behavioural and univariate results (i.e., 4- and 12-channel vocoded speech, **low sensory noise** and **high sensory noise**; names of fitted parameters are highlighted as shown). This is sufficient to allow our model to simulate the overall accuracy of perception (though not the fine-grained pattern of perceptual confusions, which is beyond the scope of the present simulation). We note that the similarity of these simulated degraded feature representations resembles the similarity of the acoustic forms of the vocoded spoken words.

The two prior knowledge conditions (Neutral and Match) were differentiated by prior lexical expectations, i.e., the prior probability of each of the 24 words in the models vocabulary. The prior expectation for the Neutral condition was defined as a uniform distribution over all the words in the set (i.e., each word was assigned a prior probability of 0.042 equivalent to 1/24). For the Match condition, the prior probability was determined by the probability of hearing matching speech after a written word was presented. In the experiment overall, there were 288 match trials and 48 mismatch trials; hence, a prior of 0.857 for the specific written word that was presented. However, because the written word was not always remembered correctly by participants, we multiplied this probability by behavioural performance in the “written only” condition (82.14% correct on average) to estimate the prior probability of the matching word and made all other words equally probable, such that the summed activation of lexical units was 1. Lexical expectations in the Neutral and Match conditions were transformed from the lexical level into phonetic feature expectations by multiplication of lexical probabilities by the word-to-feature transformation matrix.

A simulated word recognition trial in both models began by specifying the prior lexical knowledge for a Match or a Neutral trial at the output and presenting a degraded speech representation for one of the 24 words to the input (both as described above). Based on these initial activation values, an iterative updating process operated to combine prior knowledge and sensory input until a **stopping criterion** (defined on the basis of changes in lexical activation) or until a maximum number of iterations was performed. For both the Sharpened Signal and Prediction Error models, the maximum number of iterations was set to 500.

#### Sharpened signal model

In the Sharpened Signal model, sensory input that corresponds to expected words is enhanced and therefore plays a greater role in updating lexical activation values. This was achieved by generating a sharpened feature representation by multiplying the observed sensory input (over a set of features, i = 1:37) by a representation of the expected sensory input (derived from the set of expected words, w = 1:24).

First, the expected word was transformed from a lexical representation into a feature representation:

$$\mathrm{expected features}\;\left(i\right)=\mathrm{prior word}\;\left(w\right)\;*\;\mathrm{feature}\text{-}\mathrm{to}\text{-}\mathrm{word matrix}\;{\left(w,i\right)}^{T}$$

Then, the expected features were used to enhance the expected part of the sensory input:

sharpened features(i)=sensory input(i)*(1+expected features(i))

This sharpened set of phonetic features was then normalized and combined with the sensory input to form the input for the next iteration. An **update weight** parameter was fitted for the Sharpened Signal model (the same for all words and noise levels) to determine how much the sensory representation changed in each iteration.

updated sharpened features(i)=sensory input(i)+(update weight*sharpened features(i))

These sharpened features were then transformed to generate an updated word representation:

updated word(w)=updated sharpened feature(i)*feature-to-word matrix(w,i)

Iterations continued until a single lexical item became more strongly activated than any other item at the output based on a stopping criterion parameter, based on the difference between the maximum word value and the mean plus one standard deviation of all word activation values:

if max(updated word(w))-(mean(updated word(w))+standard deviation(updated word(w)))>stoppingcriterion

This **stopping criterion** parameter was fitted for the Sharpened Signal model and was the same for all words and noise levels.

#### Prediction error model

In the Prediction Error model, Prediction Errors were computed by comparing the heard sensory features (i = 1:37) with sensory features derived from the expected word (w = 1:24).

First, the expected word was transformed from a lexical representation to a feature representation:

$$\mathrm{expected features}\;\left(i\right)=\mathrm{prior word}\;\left(w\right)\;*\;\mathrm{feature}\text{-}\mathrm{to}\text{-}\mathrm{word matrix}\;{\left(w,i\right)}^{T}$$

Then, the expected features were used to explain away the expected part of the sensory input:

prediction error features(i)=sensory input(i)–expected features(i)

Based on this, the feature prediction error was transformed into a word prediction error:

prediction error word(w)=prediction error features(i)*feature-to-word matrix(w,i)

From this, an updated word representation can be computed by adding the word prior to the word prediction error multiplied by an **update weight** (equivalent to that used in the Sharpened Signal model), and a precision value:

updated word(w)=word prior(w)+(update weight*precision*prediction error word(w))

The “update weight” parameter was fitted for the Prediction Error model and was the same for all words and noise levels. The precision of the Prediction Error was determined for each word and noise level by combining the precisions of its constituents

$$\begin{array}{l} \mathrm{precision}=\;\mathrm{standard deviation}\left(\mathrm{word prior}\;\left(w\right)\right)\;/\;\mathrm{sum}\left(\mathrm{word prior}\;\left(w\right)\right)+\\ \;\mathrm{standard deviation}\left(\mathrm{sensory input}\;\left(i\right)\right)\;/\;\mathrm{sum}\left(\mathrm{sensory input}\;\left(i\right)\right)). \end{array}$$

Iterations continued until the prediction error was smaller than a **stopping criterion**:

if sum(abs(prediction error word(w)))<stopping criterion

This **stopping criterion** parameter was fitted for the Prediction Error model and was the same for all words and noise levels.

#### Relating model output to behavioural and fMRI measures

Several different measures can be derived from the operation of these computational models, which we used to simulate the behavioural, univariate, and multivariate fMRI results.

To simulate the behavioural performance, we tested whether each word presented was correctly identified by the model based on the state of the lexical representations at the end of the iterative update process (i.e., the posterior word representation). These output representations were transformed into probabilities using a softmax transfer function with a **temperature** parameter, fitted independently for Sharpened Signal and Prediction Error simulations to determine the degree of competition between active words. To simulate inconsistent or uncertain behavioural responses, we added Gaussian random noise to the word probabilities with the amount of noise determined by a **behavioural noise** parameter (again, fitted independently for each simulation Sharpened Signal and Prediction Error) and selected the word with the highest value as the response. The addition of random noise simulates word reports as resulting from additional “noisy” processes that follow computation of the likely word candidates (e.g., memory, attention, motor mapping that in turn influence how precepts lead responses). Based on whether the word chosen matches the word presented, we can calculate the word recognition performance of the model.

To simulate the univariate fMRI results, we counted the number of iterations the models needed to satisfy the **stopping criterion** (as described for each simulation). Our reasoning was that the number of processing iterations in the model serves as a proxy for the duration of the word recognition process and that, all other things being equal, a longer period of neural processing should lead to an increased BOLD signal during identification of a spoken word (see [63,112] for further discussion). This is not to say that other differences between conditions equated for processing time would not also give rise to differences in the BOLD response; only that, all other things being equal, longer processing time will lead to an increased BOLD response. Furthermore, this outcome measure (unlike, for instance, Prediction Error) is common to both sets of simulations.

To simulate the multivariate fMRI results, we tested the similarity of the sharpened feature and prediction error feature representations after the first model iteration (for the Sharpened Signal and Prediction Error model, respectively). We decided to use representations from the first iteration because we did not want to make further assumptions for how these signals are integrated over time that might favour one model or other (because Sharpened Signal and Prediction Error models show different settling dynamics) or that differentially impact one or more experimental condition (since settling dynamics may also differ between conditions). We leave it to later work using temporally sensitive neural measures (such as MEG or eCog) to explore how settling dynamics impact on neural representations of speech content. Similarly, to ensure that Sharpened Signal and Prediction Error models are more comparable, we removed the sign of the Prediction Error signal such that multivariate analyses are always performed on positive feature representations in both models. We assumed that these feature representations (or, equivalently, these representations multiplied by the feature-to-word matrix) can serve as a surrogate for multi-voxel patterns of searchlights in our posterior STS ROI. To simulate the influence of measurement noise on measured fMRI responses and, hence, multivariate similarity measures, we added a noise pattern to each of the activation patterns prior to computing correlations between feature representations. Specifically, we added Gaussian noise (with a standard deviation of 2 for both simulations) to the sharpened feature and prediction error feature representations before we conducted RSA. For each computational model, we then computed a dissimilarity matrix (based on a 1–Pearson correlation) for the feature representations for all word pairs in the model simulation of all four conditions. We then used this observed RDM and applied the same hypothetical model RDMs used in the multivariate analysis of the fMRI data (i.e., greater similarity for word-pairs within each triple that share the same vowel compared to words in different triples with different vowels). This comparison was conducted separately for each of the four key experimental conditions (Neutral/Matching priors, 4- and 12-channel speech), and Fisher-z-transformed similarity values were computed as for the fMRI data.

#### Model fitting procedure

We used a standard non-linear optimisation procedure implemented in Matlab (fminsearch, Matlab, The MathWorks, Inc.) to separately fit the following six parameters for the Sharpened Signal and Prediction Error models: (1) **low sensory detail**: the level of noise added to simulate 4-channel speech; (2) **high sensory detail**: the level of noise added to simulate 12-channel speech; (3) **update weight**: the amount by which prior representations are updated during a single processing iteration; (4) **stopping criterion**: the measure computed to determine when the iterative model process converged; (5) **temperature** parameter: this determined the degree of winner-take-all competition during response selection; (6) **behavioural noise**: simulating the degree of uncertainty and guessing in model responses. Sensitivity analyses (S3 Fig) show that parameters (1) and (2) influence both behavioural outcomes and univariate responses, parameters (3) and (4) largely influence settling time (and, hence, univariate responses), and parameters (5) and (6) influence behavioural outcomes.

The models were fitted to minimise the sum-squared error difference between model outcomes and observed behavioural and univariate results averaged over participants (see Fig 3). The analyses reported here constitute fixed-effects analyses, because we fitted the models to the group means. Specifically, we computed the sum of two error terms to quantify the difference between the model prediction and observed data. First, we computed the difference between the behavioural performance predicted by the model and the actual behaviour in the four conditions. Second, we computed the difference between the univariate results predicted by the model and the actual univariate results in the posterior STS. To relate model iterations to BOLD signal estimates, we normalized the univariate fMRI results (by dividing the extracted beta values in each condition by the maximum beta value) and the model outcome (by dividing the number of iterations by the maximum possible number of iterations, i.e., 500). Because simulations of both behavioural and fMRI responses were prone to chance variation (due to the influence of the various sources of added noise described above) we used the average results of 10 replications for each condition and item when computing and optimising model fit. The resulting six model parameters for the Sharpened Signal model were [0.2456, 0.4094, 0.002022, 2.198, 2.556, 0.01057], and the model parameters for the Prediction Error model were [0.3559, 0.5825, 0.03414, 0.407, 1.327, 0.00281].

#### Model fit evaluation

Due to the presence of several sources of random noise in the simulation model, we used Monte-Carlo methods to evaluate the goodness of fit of the two models to the data. We used the optimal parameters listed above to compute the distribution of model outcomes for 1,000 replications of each condition and item from the experiment. From these distributions, we could observe the likelihoods of the data given each model simulating (1) the behavioural results, (2) the mean parameter estimates in the left posterior STS from the univariate fMRI analysis, and (3) multivariate results for the left posterior STS.

These likelihoods were fit with a 1-dimensional kernel estimation function (kdensity function in Matlab) with a width specified based on visual inspection of the individual empirical probability density functions (kernel width set to 0.1 for all simulations and conditions). We fitted these kernel density estimates for the observed data for each model, condition, and data type. We then combined the density estimates over the four conditions for each model and data type by computing joint probabilities (i.e., the products of the four kernel density estimates of each condition). Essentially the same results were obtained by fitting four-dimensional kernel densities over all four conditions simultaneously. We computed the evidence ratio of Akaike weights to estimate how much support the data provides in favour of the Prediction Error over the Sharpened Signal model. Because both models have the same number of free parameters, the ratio of the Akaike weights can be directly calculated by $\frac{Likelihood\;Predictive\;Coding\;model}{Likelihood\;Sharpening\;model}$ [64].

## Supporting Information

S1 Fig Effect of mismatching prior expectations. **(A)** Behavioural results. **(B)** Univariate results: Main effect of prior knowledge (Matching versus Mismatching Prior) depicted on a rendered brain (*p* < 0.05 voxelwise FWE, n = 21). **(C)** Mean beta values extracted from the independent region of interest in the posterior STS [57] illustrate reduced BOLD signal during Match conditions (solid black) in contrast to Neutral (white) and Mismatch (green) conditions. Error bars indicate standard error of the mean after between-subject variability has been removed suitable for repeated measures comparisons [62]. Please refer to S1 Data at https://osf.io/2ze9n/ (doi: 10.17605/OSF.IO/2ZE9N) for the numerical values underlying these figures.
(TIF)

S2 Fig **Network architecture and example representations for (A) Sharpened Signal and (B) Prediction Error models.** Common components of both models are outlined in black. Differences between the two models are coloured in orange (Sharpened Signal) and blue (Prediction Error). Both models map from a feature-based representation of consonant-vowel-consonant symbols that have been degraded by the addition of random, probabilistic noise within the different groups of units representing specific feature types (place, manner, voicing, etc.). Input for the word “thing” is shown for both models, using representations degraded to simulate 4-channel and 12-channel noise vocoded speech (based on clarity parameters fit for each of the simulations). A clear speech (un-degraded) representation of the word “thing” is shown for comparison, though this wasn’t presented to either model. Hinton diagrams show the activation of each individual unit with the area of the squares proportional to activation values or probabilities, supplemented by colour scales as shown. In both models, lexical representations are specified over a bank of 24 localist units (one for each word in the models’ vocabulary and experimental item set). These lexical representations are initialised to express the prior probability of each word being presented based on prior written text (“THING,” Match condition) or a neutral string (“XXXX,” Neutral condition). In both models, a word-to-feature matrix links words to their constituent phonetic features and a feature-to-word matrix links phonetic features to words (these two matrices are the transpose of each other). There are some key differences between the two models. In the Sharpened Signal model (A), prior knowledge is used to increase the gain of expected sensory features, such that expected features are preferentially activated in Sharpened Feature representations at the intermediate level of the model. These Sharpened Features are then used to update lexical representations. Thus, Match trials lead to Sharpened Feature representations that resemble those from speech signals with greater sensory detail. In contrast, in the Prediction Error model (B), expected sensory features are subtracted from the observed sensory input, and Prediction Error feature representations at the intermediate level are used to update lexical representations. These Prediction Error representations contain negative values (blue colours) for expected features that are presented in a degraded form; these negative prediction errors carry information concerning the identity of the speech signal in Match 4 trials that is absent for Match 12 trials in which speech is less degraded.
(TIF)

S3 Fig Sensitivity analysis. **(A) Prediction Error model. (B) Sharpened Signal model.** The blue curves illustrate how the sum squared error (SSE, *y*-axis) for model fit to the behavioural (left column), univariate fMRI (middle columns), and multivariate fMRI (right columns) data changes for a range of parameters (along the *x*-axis). Each graph therefore shows the influence of each of the six parameters: (1) low clarity, (2) high clarity, (3) prior update weight, (4) stopping criterion, (5) temperature, and (6) behavioural noise on model fit. The red dot on each graph indicates the final parameters chosen by nonlinear optimisation. Univariate and multivariate fMRI data come from ROI coordinates based on univariate analysis (Fig 3C). Please refer to S2 Data at https://osf.io/2ze9n/ (doi: 10.17605/OSF.IO/2ZE9N) for the numerical values underlying these figures.
(TIF)

S4 Fig **Representation of phonetic form in Inferior Frontal regions (A)** Univariate results: Main effect of prior knowledge (Matching versus Neutral Prior) depicted on a rendered brain (*p* < 0.05 voxelwise FWE, n = 21). White circle marks post-hoc defined clusters of interest in the left Inferior Frontal Gyrus (IFG). **(B,C)** Fisher-z-transformed Spearman correlation coefficients for each of the four conditions in two left IFG clusters (defined by the univariate analysis) show a significant correlation in the Match 4-channel condition and a significant reduction in correlation with increased sensory detail Match 4-channel compared to Match 12-channel. Error bars indicate standard error of the mean after between-subject variability has been removed, which is appropriate for repeated-measures comparisons [62]. Please refer to S1 Data at https://osf.io/2ze9n/ (doi: 10.17605/OSF.IO/2ZE9N) for the numerical values underlying these figures.
(TIF)

S5 Fig Comparison of four different, hierarchically organised hypothesis RDMs of speech perception. Left Panel: **(A)** dissimilarity of the acoustic properties of the speech stimuli used in our study (see Supplementary Methods for details), **(B)** dissimilarity of feature representation for the canonical forms of the speech provided as the input to our computational simulations, **(C)** dissimilarity of the segment representations of the word stimuli used in the experiment, scored based on the number of position-specific phonemes shared between words pairs, and **(D)** main hypothesis RDM assuming increased similarity between pairs of syllables that shared the same vowel (e.g., “sing” and “thing” should have more similar patterns than “sing” and “bath”). These RDMs can be considered to describe a hierarchy of speech representations from the fine-grained acoustic RDM to the most abstract syllable RDM used in our main analysis. These hypothesis RDMs are positively correlated with each other and hence can be considered as testing related proposals concerning neural representations of spoken words. Right panel **(E–H)** shows the results for the Kendall’s Tau A correlation coefficients (suitable for comparisons between binary and fine-grained RDMs; see Supplementary Methods for details) as extracted from the independent region of interest in the left posterior STS (pSTS, Fig 4B). Only the segment **(G)** and the syllable RDM **(H)** revealed a significant interaction of sensory detail and prior knowledge, similar to that shown in Fig 4B. Please refer to S1 Data at https://osf.io/2ze9n/ (doi: 10.17605/OSF.IO/2ZE9N) for the numerical values underlying these figures.
(TIF)

S6 Fig Cross-subject consistency based on empirical and simulated RDMs. **(A)** Empirical RDMs were extracted from the independent ROI in the left posterior STS (pSTS, Fig 4B), and the Simulated RDMs based on either **(B)** the Sharpened Signal or **(C)** the Prediction Error model were computed for 21 simulated participants. The cross-subject consistencies from the empirical RDMs and simulated RDMs from the Prediction Error model show the same crossover interaction of sensory detail and prior knowledge shown before (Fig 4B–4D). Please refer to S1 Data at https://osf.io/2ze9n/ (doi: 10.17605/OSF.IO/2ZE9N) for the numerical values underlying these figures.
(TIF)

S7 Fig Representational similarity searchlight analysis in the whole brain. Interaction of Prior information (Match/Neutral) x Sensory detail (4- versus 12-channel) depicted on rendered brain (F-contrast, *p* < 0.001 uncorrected, k > 10 voxels; searchlight analysis with a voxel size of 3 x 3 x 3.75 mm; see S4 Table for coordinates). https://osf.io/2ze9n/ (doi: 10.17605/OSF.IO/2ZE9N).
(TIF)

S1 Table Univariate Analysis—F-contrast: Main effect Match/Neutral, *p* < 0.05 FWE (voxelwise correction) (XLS)

S2 Table Univariate Analysis—F-contrast: Main effect sensory detail, *p* < 0.05 FWE (voxelwise correction) (XLS)

S3 Table Univariate Analysis—F-contrast: Prior information (Match/Neutral) x Sensory detail full interaction, *p* < 0.001 uncorrected, k > 10 voxels (XLS)

S4 Table Univariate Analysis—F-contrast: Main effect Match/Mismatch, *p* < 0.05 FWE (voxelwise correction) (XLS)

S5 Table RSA—F-contrast: Prior information (Match/Neutral) x Sensory detail full interaction, *p* < 0.001 uncorrected, k > 10 voxels (searchlight analysis with a voxel size of 3 x 3 x 3.75 mm) (XLS)

S1 Text Text file describing the supplementary methods and supplementary results and discussion. (DOCX)

## Abbreviations

fMRI: functional magnetic resonance imaging
IFG: inferior frontal gyrus
RDM: representational dissimilarity matrix
ROI: region of interest
RSA: representational similarity analysis
STG: superior temporal gyrus
STS: superior temporal sulcus
