# Supplementary figures and images for: Prediction Errors but Not Sharpened Signals Simulate Multivoxel fMRI Patterns during Speech Perception
Source: PLoS Biol. 2016 Nov 15;14(11):e1002577. doi: 10.1371/journal.pbio.1002577 (PMC5112801; doi:10.1371/journal.pbio.1002577)

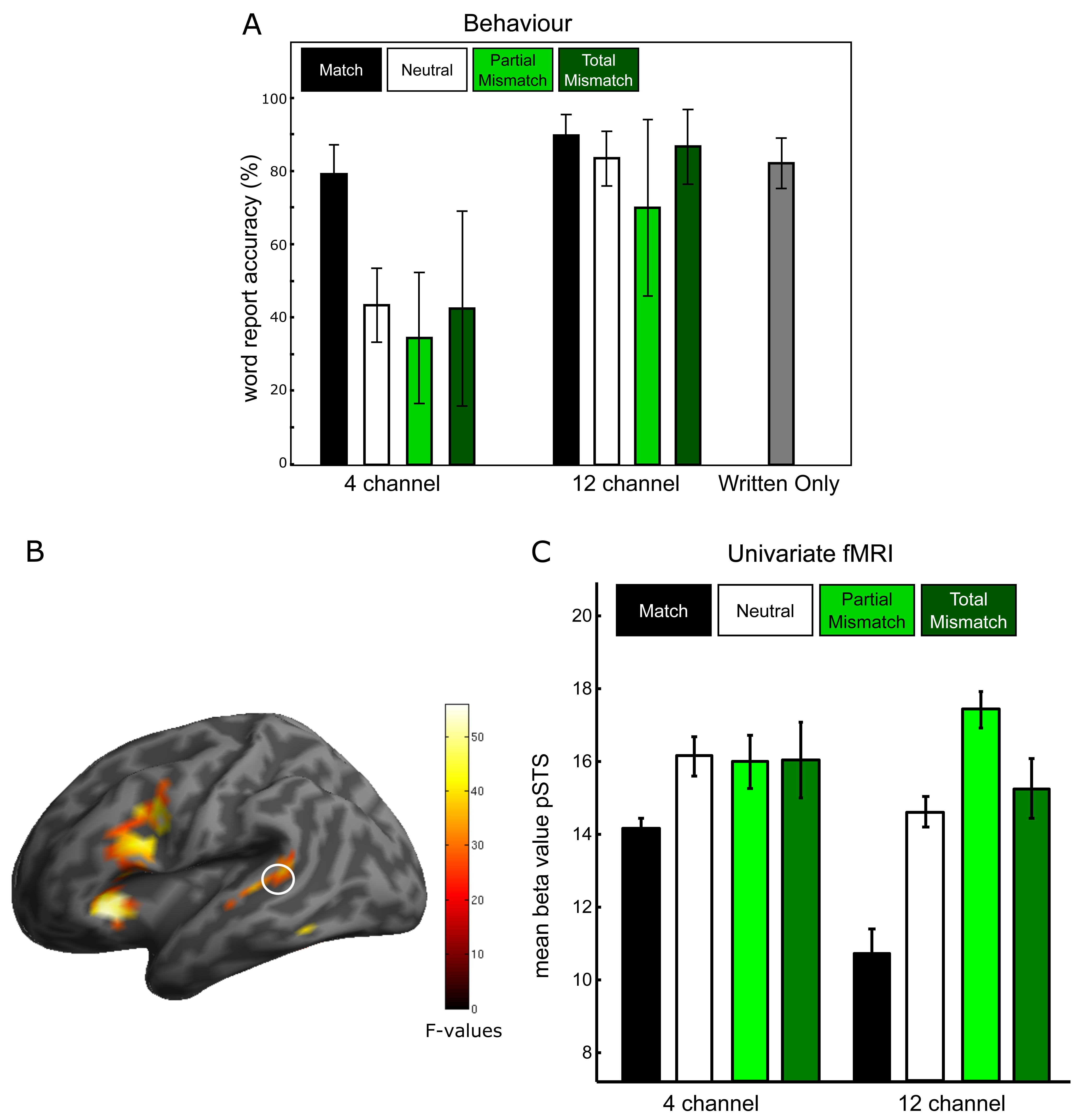

Supplement: S1 Fig — (A) Behavioural results. (B) Univariate results: Main effect of prior knowledge (Matching versus Mismatching Prior) depicted on a rendered brain (p < 0.05 voxelwise FWE, n = 21). (C) Mean beta values extracted from the independent region of interest in the posterior STS [57] illustrate reduced BOLD signal during Match conditions (solid black) in contrast to Neutral (white) and Mismatch (green) conditions. Error bars indicate standard error of the mean after between-subject variability has been removed suitable for repeated measures comparisons [62]. Please refer to S1 Data at https://osf.io/2ze9n/ (doi: 10.17605/OSF.IO/2ZE9N) for the numerical values underlying these figures. (TIF) [file pbio.1002577.s001.tif]

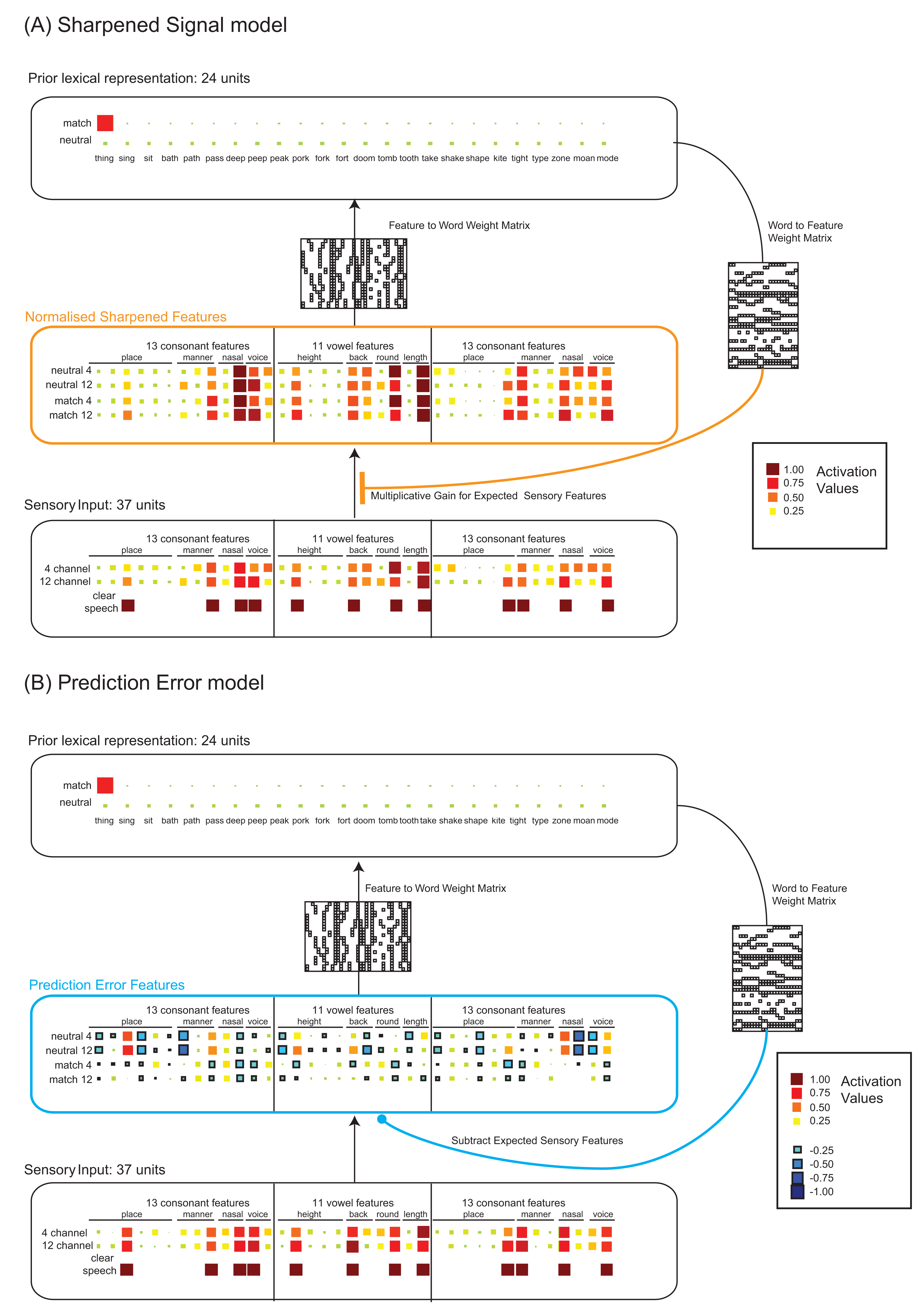

Supplement: S2 Fig — Network architecture and example representations for (A) Sharpened Signal and (B) Prediction Error models. Common components of both models are outlined in black. Differences between the two models are coloured in orange (Sharpened Signal) and blue (Prediction Error). Both models map from a feature-based representation of consonant-vowel-consonant symbols that have been degraded by the addition of random, probabilistic noise within the different groups of units representing specific feature types (place, manner, voicing, etc.). Input for the word “thing” is shown for both models, using representations degraded to simulate 4-channel and 12-channel noise vocoded speech (based on clarity parameters fit for each of the simulations). A clear speech (un-degraded) representation of the word “thing” is shown for comparison, though this wasn’t presented to either model. Hinton diagrams show the activation of each individual unit with the area of the squares proportional to activation values or probabilities, supplemented by colour scales as shown. In both models, lexical representations are specified over a bank of 24 localist units (one for each word in the models’ vocabulary and experimental item set). These lexical representations are initialised to express the prior probability of each word being presented based on prior written text (“THING,” Match condition) or a neutral string (“XXXX,” Neutral condition). In both models, a word-to-feature matrix links words to their constituent phonetic features and a feature-to-word matrix links phonetic features to words (these two matrices are the transpose of each other). There are some key differences between the two models. In the Sharpened Signal model (A), prior knowledge is used to increase the gain of expected sensory features, such that expected features are preferentially activated in Sharpened Feature representations at the intermediate level of the model. These Sharpened Features are then used to update lexical represent [file pbio.1002577.s002.tif]

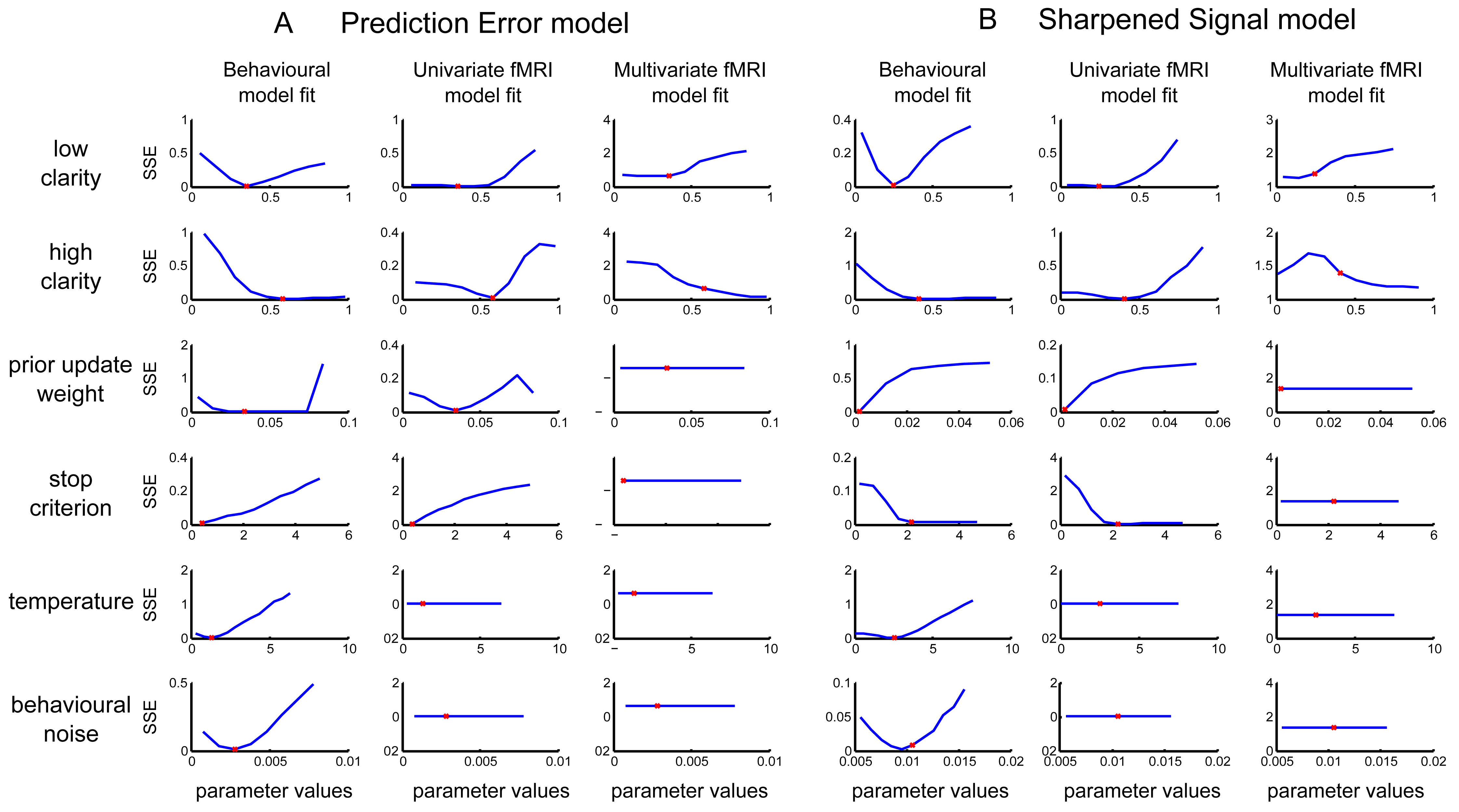

Supplement: S3 Fig — (A) Prediction Error model. (B) Sharpened Signal model. The blue curves illustrate how the sum squared error (SSE, y-axis) for model fit to the behavioural (left column), univariate fMRI (middle columns), and multivariate fMRI (right columns) data changes for a range of parameters (along the x-axis). Each graph therefore shows the influence of each of the six parameters: (1) low clarity, (2) high clarity, (3) prior update weight, (4) stopping criterion, (5) temperature, and (6) behavioural noise on model fit. The red dot on each graph indicates the final parameters chosen by nonlinear optimisation. Univariate and multivariate fMRI data come from ROI coordinates based on univariate analysis (Fig 3C). Please refer to S2 Data at https://osf.io/2ze9n/ (doi: 10.17605/OSF.IO/2ZE9N) for the numerical values underlying these figures. (TIF) [file pbio.1002577.s003.tif]

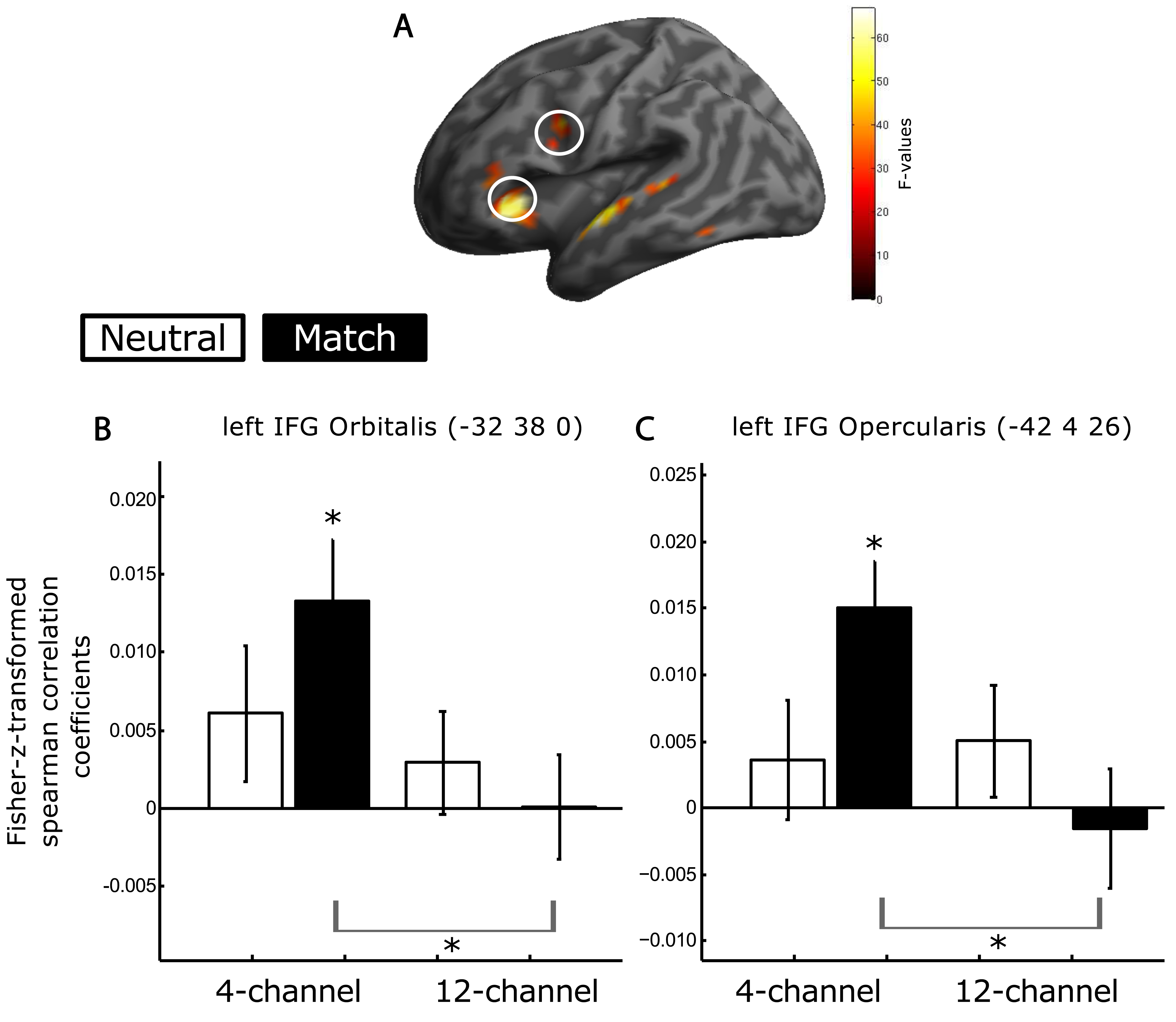

Supplement: S4 Fig — Representation of phonetic form in Inferior Frontal regions (A) Univariate results: Main effect of prior knowledge (Matching versus Neutral Prior) depicted on a rendered brain (p < 0.05 voxelwise FWE, n = 21). White circle marks post-hoc defined clusters of interest in the left Inferior Frontal Gyrus (IFG). (B,C) Fisher-z-transformed Spearman correlation coefficients for each of the four conditions in two left IFG clusters (defined by the univariate analysis) show a significant correlation in the Match 4-channel condition and a significant reduction in correlation with increased sensory detail Match 4-channel compared to Match 12-channel. Error bars indicate standard error of the mean after between-subject variability has been removed, which is appropriate for repeated-measures comparisons [62]. Please refer to S1 Data at https://osf.io/2ze9n/ (doi: 10.17605/OSF.IO/2ZE9N) for the numerical values underlying these figures. (TIF) [file pbio.1002577.s004.tif]

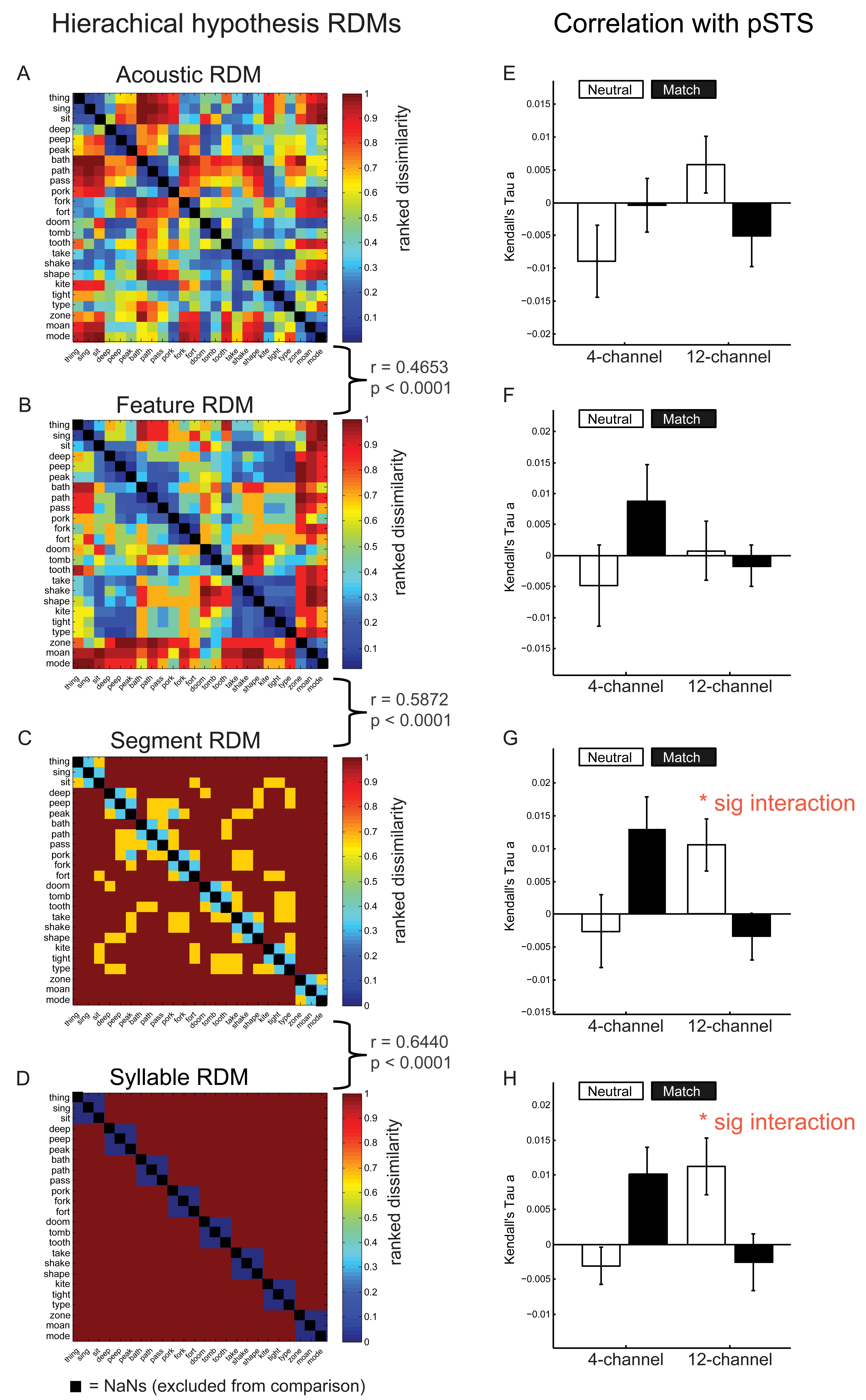

Supplement: S5 Fig — Left Panel: (A) dissimilarity of the acoustic properties of the speech stimuli used in our study (see Supplementary Methods for details), (B) dissimilarity of feature representation for the canonical forms of the speech provided as the input to our computational simulations, (C) dissimilarity of the segment representations of the word stimuli used in the experiment, scored based on the number of position-specific phonemes shared between words pairs, and (D) main hypothesis RDM assuming increased similarity between pairs of syllables that shared the same vowel (e.g., “sing” and “thing” should have more similar patterns than “sing” and “bath”). These RDMs can be considered to describe a hierarchy of speech representations from the fine-grained acoustic RDM to the most abstract syllable RDM used in our main analysis. These hypothesis RDMs are positively correlated with each other and hence can be considered as testing related proposals concerning neural representations of spoken words. Right panel (E–H) shows the results for the Kendall’s Tau A correlation coefficients (suitable for comparisons between binary and fine-grained RDMs; see Supplementary Methods for details) as extracted from the independent region of interest in the left posterior STS (pSTS, Fig 4B). Only the segment (G) and the syllable RDM (H) revealed a significant interaction of sensory detail and prior knowledge, similar to that shown in Fig 4B. Please refer to S1 Data at https://osf.io/2ze9n/ (doi: 10.17605/OSF.IO/2ZE9N) for the numerical values underlying these figures. (TIF) [file pbio.1002577.s005.tif]

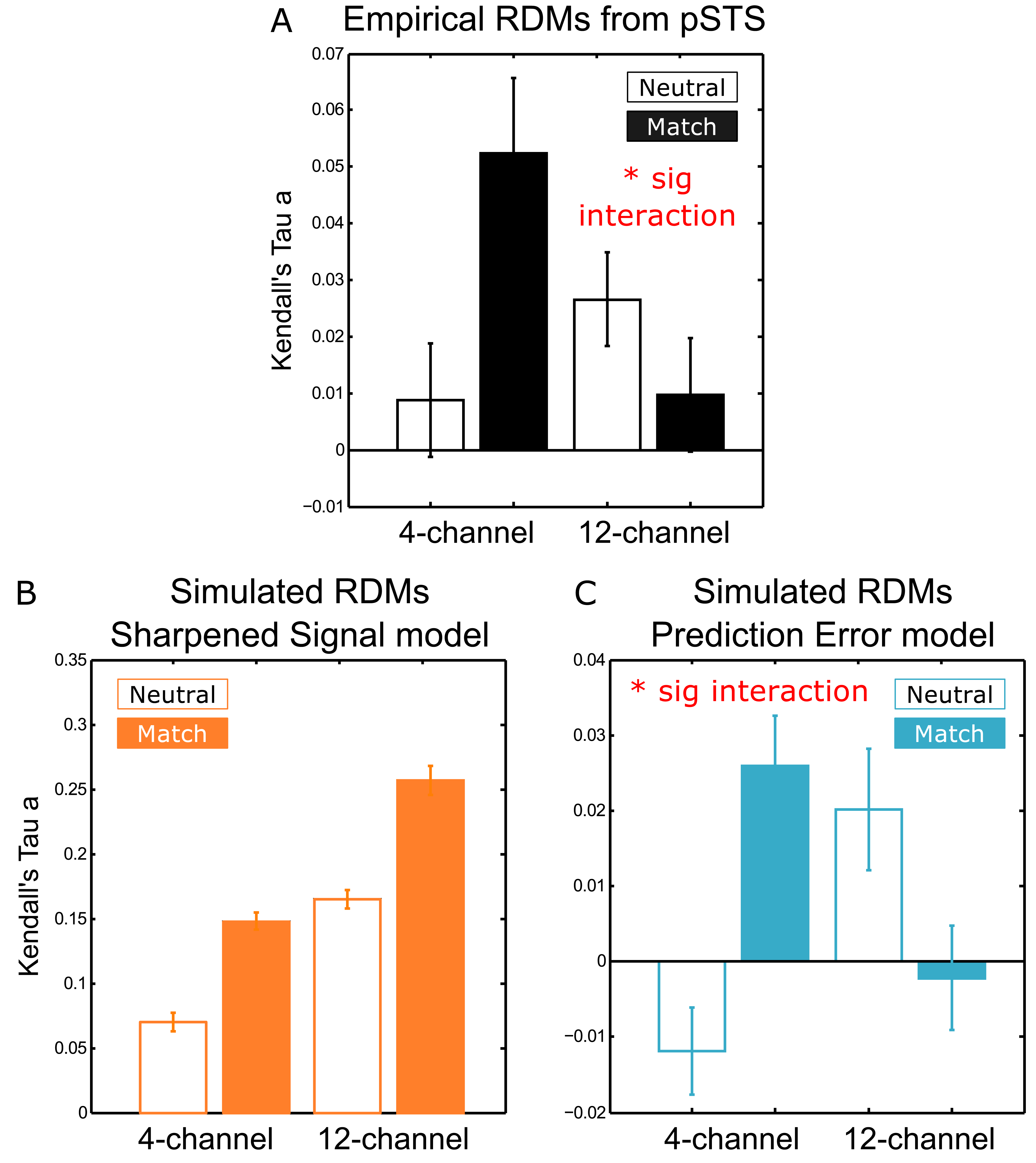

Supplement: S6 Fig — (A) Empirical RDMs were extracted from the independent ROI in the left posterior STS (pSTS, Fig 4B), and the Simulated RDMs based on either (B) the Sharpened Signal or (C) the Prediction Error model were computed for 21 simulated participants. The cross-subject consistencies from the empirical RDMs and simulated RDMs from the Prediction Error model show the same crossover interaction of sensory detail and prior knowledge shown before (Fig 4B–4D). Please refer to S1 Data at https://osf.io/2ze9n/ (doi: 10.17605/OSF.IO/2ZE9N) for the numerical values underlying these figures. (TIF) [file pbio.1002577.s006.tif]

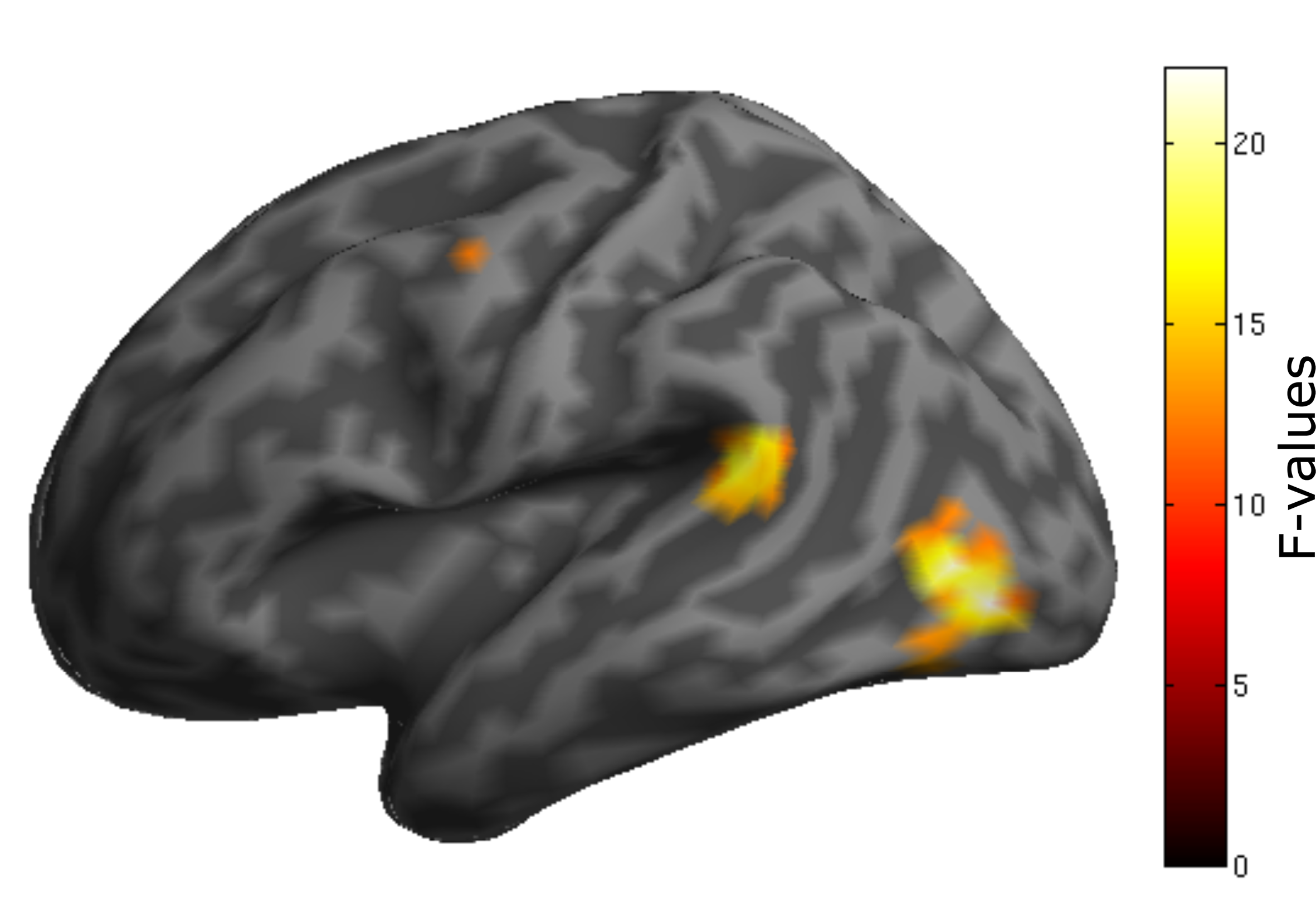

Supplement: S7 Fig — Interaction of Prior information (Match/Neutral) x Sensory detail (4- versus 12-channel) depicted on rendered brain (F-contrast, p < 0.001 uncorrected, k > 10 voxels; searchlight analysis with a voxel size of 3 x 3 x 3.75 mm; see S4 Table for coordinates). https://osf.io/2ze9n/ (doi: 10.17605/OSF.IO/2ZE9N). (TIF) [file pbio.1002577.s007.tif]
