# Supplementary material for: Prediction Errors but Not Sharpened Signals Simulate Multivoxel fMRI Patterns during Speech Perception
Source: PLoS Biol. 2016 Nov 15;14(11):e1002577. doi: 10.1371/journal.pbio.1002577 (PMC5112801; doi:10.1371/journal.pbio.1002577)
Supplement: S1 Text — (DOCX) [file pbio.1002577.s013.docx]

# Supplementary Material

Prediction errors but not sharpened signals simulate multivoxel fMRI patterns during speech perception - H Blank, H & M H Davis

# Supplementary Methods

## Comparison of four different, hierarchically organised hypothesis RDMs of speech perception.

We tested three additional hypothesis Representational Dissimilarity Matrices (RDMs) relating to (1) the acoustic properties of the speech stimuli used in our study, (2) the feature representation as used as input to our computational simulations, and (3) the segmental representation of the word stimuli, as scored based on the number of shared phonemes (SI Fig 5 left column). We will describe how these additional similarity matrices were generated in turn.

For the acoustic properties we computed acoustic (dis)similarity between pairs of speech tokens using methods described by Billig and colleagues (1). Specifically, we generated a Gammatone-based spectro-temporal representation for each speech token. A spectral dissimilarity matrix was then generated between pairs of spectro-temporal representations tokens by computing 1 minus the sample linear correlation between log-scaled spectra at all time slices. Next, the maximum-dissimilarity path through this spectral-dissimilarity matrix was found using dynamic time warping. Summed dissimilarity values along this path were computed and rescaled (dissimilarity / maximum dissimilarity) such that two identical sound files were assigned a score of 0 and the two most dissimilar sound files given a score of 1. Note that as reported by (1) greatest similarity is seen for pairs of syllables that contain the same vowel. The gammatone representation and dynamic time warping were performed using Matlab implementations of standard algorithms written by Dan Ellis (downloaded from <http://www.ee.columbia.edu/ln/rosa/matlab/>).

For the feature properties we used the input representation of speech used in our computational simulations (for details see Materials and Methods in the Main Text). A feature dissimilarity matrix was then generated by computing 1 minus the sample linear correlation between pairs of feature representations for all 24 words used in our experiment and simulations.

For the segmental properties we counted the common segments of all word pairs based on the phonemic transcription of each word (CELEX Database). This score based on the number of shared phonemes ranged from 0 to 3, because each word consisted of three segments. The common segments were transformed to a dissimilarity value by [(3 – number of common segments) / 3].

The three additional RDMs included more fine-grained similarity values (e.g., Acoustic RDM) compared to the binary RDM used in the main analysis (i.e., Syllable RDM). To not favour a simplified RDM with tied ranks (such as Syllable RDM) we repeated the RSA searchlight analysis with Kendall’s Tau A (instead of Spearman correlation). Kendall’s Tau A is more likely than Spearman correlation coefficient to prefer the true RDM over a simplified RDM containing tied ranks (2).

## Estimation of cross-subject consistency and maximum possible correlation of the observed RDM in left posterior STS

To provide an estimate of the maximum possible correlation value between the observed RDM and the hypothesized RDMs, we used the procedure described in (2) for computing the upper bound of the noise ceiling of the observed RDMs for the fMRI data. Specifically, the rank-transformed single-subject RDMs were averaged and we used in an iterative procedure to find the RDM with the maximum average correlation to the single subject RDMs (using published code from (2)).

In addition, to provide an estimate of the expected correlation value between the observed RDM and the hypothesized RDMs, given the degree of inter-subject variation in the fMRI data, we computed the cross-subject consistency of the observed RDMs (using the procedure described for computing the lower bound of the noise ceiling in (2) and the corresponding published code). Specifically, we used a leave-one-subject-out procedure in which we correlated (using Kendall's Tau A coefficient) each subject’s empirically observed RDM with the mean observed RDM of the remaining 20 subjects, separately for the four conditions. Then we computed the mean over these correlation values for each condition to estimate an empirically-derived hypothesis RDM for similarity between the word stimuli used in our experiment. The empirical RDMs were computed within an 8 mm sphere (corresponding to the 8 mm sphere used in the whole-brain searchlight analysis) centred on the voxel specified for the independent ROI in the left posterior STS (3). To compare this empirical cross-subject consistency with the expected cross-subject consistency based on the Prediction Error and Sharpened Signal models, we performed the same leave-one-subject-out correlation analysis on single subject RDMs for 21 simulated participants (i.e., treating individual simulation runs as individual participants). We increased the amount of Gaussian noise added to the prediction error and sharpened signal representations (5 standard derivations) so that overall similarity was comparable for empirical and simulated RDMs. Importantly, the same amount of noise was added to all four conditions and to both models.

# Supplementary Results and Discussion

## Comparison of responses following mismatching written text

### Behavioural Analysis.

We confirmed that providing informative prior expectations improves perception of degraded speech in comparison to both providing neutral or mismatching prior information **(**SI Fig 1 A)**.** A two-way repeated measures ANOVA with the factors sensory detail (4- vs. 12-channel) and prior knowledge (Match vs. Neutral vs. Mismatch) revealed significant main effects of sensory detail on word report (*F*(1, 20) = 139.988, *p* < 0.001, eta squared = 87.50) and prior knowledge (*F*(1, 20) = 80.652, *p* < 0.001, eta squared = 80.13), and a significant interaction (*F*(1, 20) = 14.617, *p* < 0.001). Post-hoc paired t-tests revealed that word report for degraded speech that mismatched with prior text was less accurate than for speech that matched prior text for both the 4-channel (*t*(20) = 8.343, *p* < 0.001) and the 12-channel conditions (*t*(20) = 4.590, *p* < 0.001). However, word report did not differ between Mismatch and Neutral condition at either level of sensory detail (4-channel: *t*(20) = 1.71, *p* = 0.102; 12-channel: *t*(20) = 1.531, *p* = 0. 141). This suggests that differences between Match and Neutral trials reflect the facilitatory perceptual effect of matching prior knowledge, rather than any non-specific effect of hearing degraded words after reading a written text cue.

### Univariate Results.

We sought to localise the univariate BOLD activity decrease for degraded spoken words that follow matching written words relative to words following mismatching cues (SI Fig 1 B/C). We conducted a repeated measures ANOVA with two factors: prior knowledge (Match vs. Mismatch) and level of sensory detail (4- vs. 12-channel) to assess the main effect of prior information a whole brain analysis. We collapsed across both types of mismatching conditions (partial and total mismatch; e.g., ‘shape’ - ‘shake’ and ‘shape’ - ‘zone’, respectively) between written and spoken words to increase the number of trials that could be included in this analysis. The magnitude of the BOLD responses in the left posterior STS in the Mismatch condition resembles the magnitude of the BOLD responses in the Neutral condition (SI Fig 1 C). This confirms that reduced activity observed for degraded speech that matches previously written words (compared to speech following Neutral text “XXXX”) is due to the facilitatory effect of hearing degraded speech that matches prior knowledge rather than a generic modulation of auditory responses following written text. Increased activity for speech that follows mismatching in comparison to matching written words also confirms that the difference found for Neutral > Match is not due to changes in “attention”, or baseline activation following written text since decreased activity for Match trials is not specific to a comparison with responses following uninformative cues in the Neutral condition.

## Representations of phonetic form in Inferior Frontal Regions

To provide a more complete picture of our data, we used the two regions in the Inferior Frontal Gyrus as identified by the univariate analysis on prior expectation (responses greater following Neutral than following Matching text, SI Fig 4 + SI Table 1). These regions are potentially of interest because these regions have been proposed to contribute to (predictive) processing, in particular for speech heard in adverse listening conditions (4-6). Multivariate pattern analysis has further shown that inferior frontal and adjacent precentral gyrus regions represent the identity, but not the acoustic form of heard syllables (3, 7, 8), particularly if speech is degraded.

For these two regions, we conducted ROI analyses of multivariate information content in each of our four experimental conditions. Specifically, we conducted a Repeated Measures ANOVA with factors sensory detail (4- vs. 12-channel) and prior knowledge (Match vs. Neutral). Fisher-z-transformed correlation coefficients extracted from either of the regions of interest in the IFG (Orbitalis: 623 voxels, peak MNI: x = -32, y = 38, z = 0 and Opercularis: 164 voxels peak at MNI: x = -42, y = 4, z = 26; SI Fig 4) did not reveal any significant main effect or interaction (Main effect Prior: *F* < 1 for both ROIs; Main effect sensory detail: *F*(20) = 3.965, *p* = 0.060; *F*(20) = 2.768, *p* = 0.112; Interaction: *F*(20) = 1.255, *p* = 0.276; F(20) = 3.728, *p* = 0.069; for left IFG Orbitalis; left IFG Opercularis, respectively). Post-hoc t-tests in both regions revealed a significant correlation only in the Match 4-channel condition (*t*(20) = 2.709, *p* = 0.007; *t*(20) = 3.682, *p* < 0.001) and for the paired t-test of Match 4-channel vs. Match 12 (*t*(20) = 2.268, *p* = 0.017; *t*(20)= 2.726, *p* = 0.007; for left IFG Orbitalis; left IFG Opercularis, respectively). This result for the Match 4-channel in the IFG is particularly interesting because the IFG has previously been suggested as the source of top-down predictions when written text informs the perception of degraded speech (4, 6). Furthermore, these top-down mechanisms seem to be especially important for perceptual learning observed when matching prior expectations can be used to guide perception of highly degraded speech (5, 9, 10).

## Comparison of four different, hierarchically organised hypothesis RDMs of speech perception.

The similarity values computed in the three additional RDMs are positively correlated (Acoustic to Feature RDM: *r* = 0.4653, *p* < 0.0001; Feature to Segment RDM: *r* = 0.5872; *p* < 0.0001). Importantly, the most abstract segment level description is also highly correlated with the similarity matrix constructed on the basis of the syllable triples used in the experiment (Segment to Syllable RDM = 0.6440, *p* < 0.0001, see also correlations for Acoustic to Syllable RDM: *r* = 0.3549, *p* < 0.0001; Feature to Syllable RDM: *r* = 0.4331, *p* < 0.0001). This indicates that acoustic, feature, segment, and syllable characteristics of the word stimuli used in our experiment are related to each other. Further evidence for these being a hierarchy of representations comes from comparisons between these correlations tested using one-sided t-tests for dependent correlations (11). We see significantly higher correlations between Segment to Syllable than Feature to Syllable RDMs (*t*(273) = 4.912, *p* < 0.001), a trend for higher correlations between Feature to Syllable than Acoustic to Syllable RDMs (*t*(273) = 1.379, *p* = 0.085) and significantly higher correlations between Feature to Segment than Acoustic to Segment RDMs (*t*(273) = 4.189, *p* < 0.001). This suggests a hierarchy from acoustic representations to syllable representations in the order shown in SI Fig 5.

However, results confirm that only the Segment and the Syllable hypothesis RDMs show the interaction of sensory detail and prior knowledge in the STS (Syllable RDM: *F*(1,20) = 9.302, *p* = 0.006; Segment RDM: *F*(1,20) = 6.237, *p* = 0.021; main effects were not significant for either RDM: *F*(1,20) < 0.1). There were no significant main effects or interactions of sensory detail and prior knowledge for either the Acoustic or the Feature RDM (all effects: *p* > 0.05). This result is in line with previous findings that categorical, segmental representations are an important organizing principle in STG/STS regions (12, 13). Recordings from fMRI (13) and intracranial high-density cortical surface arrays showed that the posterior STG represents the underlying identity of spoken syllables rather than producing a linear response to changes in spectrotemporal acoustic or phonetic cues (12).

## Cross-subject consistency and maximum possible correlation of the observed RDM in left posterior STS

The upper bound (that is, the maximum possible correlation value that could be observed in our fMRI data from the posterior STS) is very similar across the four conditions (Neutral 4-channel: 0.1488; Match 4-channel: 0.1715; Neutral 12-channel: 0.1573; Match 12-channel: 0.1481) and there is neither a significant interaction of sensory detail and prior knowledge (*F*(1,20) = 1.785), nor a main effect (Sensory detail: *F*(1,20) = 0.344; Prior knowledge: *F*(1,20) = 0.323). In all four conditions, the upper bound of the maximal possible correlation is substantially smaller than 1. This indicates limitations of our experimental data (e.g., low spatial resolution, high measurement noise and/or limited amounts of data). Nonetheless, none of these limitations differentially affect our four critical conditions and hence measurement noise or other extraneous factors cannot explain the significant interaction of sensory detail and prior knowledge seen in multivariate analyses.

The relatively small effect sizes that we have observed are common for multivariate fMRI analyses of speech stimuli. For RSA of speech perception, similar values of (Fisher-z transformed) correlation between fMRI-response based- and hypothesized similarity matrices have been observed previously (3, 8). Similarly, low classification accuracies are also common in decoding task events using Multivariate Classification of fMRI data (14, 15). Despite these small effect sizes, condition-specific differences in the observed correlations (i.e., the interaction of sensory detail and prior knowledge) provide compelling statistical support for neural representations of Prediction Error.

The obtained correlation values for the cross-subject consistency of the observed RDMs in the STS are higher than the correlation values obtained for the main RSA analysis (i.e., the correlation with Syllable RDM, compare SI Fig 5H and SI Fig 6A). This indicates that the observed correlation values in our fMRI RSA analysis are smaller than expected due to limitations of the hypothesis RDMs (2) and that there is potential for alternative hypothesis RDMs to provide higher correlation values with the observed RDMs. However, the cross-subject consistency of the observed RDMs also showed a significant cross-over interaction of sensory detail and prior knowledge (*F*(1,20) = 6.443, *p* = 0.0196) and no main effects (Sensory detail: *F*(1,20) = 0.968; Prior knowledge: *F*(1,20) = 1.298, SI Fig 6A). This suggests that the information present in multivoxel fMRI patterns differs among our four conditions even when this is tested without assuming a hypothesis RDM. Simulations show that this is in line with the Prediction Error model. Simulated cross-subject consistency from the Sharpened Signal model (SI Fig 6B) showed two significant main effects (Sensory detail: *F*(1,20) = 153.023, *p* < 0.001; Prior knowledge: *F*(1,20) = 111.232, p < 0.001), but no interaction (*F*(1,20) = 0.340). This is the same pattern as observed in the main simulation using the Syllable RDM as the hypothesis RDM (Fig 4C) which does not resemble the empirical data (i.e., Fig 4B, SI Fig 6A). In contrast, the cross-subject consistency simulated with the Prediction Error model (SI Fig 6C) showed a significant cross-over interaction of sensory detail and prior knowledge (*F*(1,20) = 15.217, *p* = 0.0009) and no main effects (Sensory detail: *F*(1,20) = 0.059; Prior knowledge: *F*(1,20) = 0.906). The reduction of simulated cross-subject consistency in both the Neutral 4-channel and the Match 12-channel conditions is explained by uninformative Prediction Errors in these conditions. This is due to either uninformative sensory information (Neutral 4-channel) or informative sensory information explained away by matching prior expectations (Match 12-channel). Again, this is the same pattern as observed in the main simulation using the Syllable RDM as the hypothesis RDM (Fig 4D) which resembles the empirical data (Fig 4B, SI Fig 6A). These cross-subject consistency measures suggest that with an appropriate hypothesis RDM we could have improved the correlation values obtained with our theoretically motivated hypothesis RDM (Syllable RDM). However, since these correlation values still differed across the four conditions our conclusions that neural representations of sensory detail and prior knowledge are in line with our Prediction Error simulation would still hold. Indeed, the good correspondence seen between simulated and observed multivariate analyses of cross-subject consistency further strengthens this conclusion.

# References

1. Billig AJ, Davis MH, Deeks JM, Monstrey J, Carlyon RP. Lexical influences on auditory streaming. Curr Biol. 2013;23(16):1585-9.

2. Nili H, Wingfield C, Walther A, Su L, Marslen-Wilson W, Kriegeskorte N. A toolbox for representational similarity analysis. PLoS Comput Biol. 2014;10(4):e1003553.

3. Evans S, Davis MH. Hierarchical Organization of Auditory and Motor Representations in Speech Perception: Evidence from Searchlight Similarity Analysis. Cerebral cortex. 2015;25(12):4772-88.

4. Sohoglu E, Peelle JE, Carlyon RP, Davis MH. Predictive Top-Down Integration of Prior Knowledge during Speech Perception. J Neurosci. 2012;32(25):8443-53.

5. Eisner F, McGettigan C, Faulkner A, Rosen S, Scott SK. Inferior frontal gyrus activation predicts individual differences in perceptual learning of cochlear-implant simulations. J Neurosci. 2010;30(21):7179-86.

6. Obleser J, Kotz SA. Expectancy constraints in degraded speech modulate the language comprehension network. Cereb Cortex. 2010;20(3):633-40.

7. Lee YS, Turkeltaub P, Granger R, Raizada RDS. Categorical Speech Processing in Broca's Area: An fMRI Study Using Multivariate Pattern-Based Analysis. J Neurosci. 2012;32(11):3942-8.

8. Du Y, Buchsbaum BR, Grady CL, Alain C. Noise differentially impacts phoneme representations in the auditory and speech motor systems. P Natl Acad Sci USA. 2014;111(19):7126-31.

9. Sohoglu E, Davis MH. Perceptual learning of degraded speech by minimizing prediction error. Proc Natl Acad Sci U S A. 2016;113(12):E1747-56.

10. Sehm B, Schnitzler T, Obleser J, Groba A, Ragert P, Villringer A, et al. Facilitation of Inferior Frontal Cortex by Transcranial Direct Current Stimulation Induces Perceptual Learning of Severely Degraded Speech. J Neurosci. 2013;33(40):15868-78.

11. Steiger JH. Tests for Comparing Elements of a Correlation Matrix. Psychol Bull. 1980;87(2):245-51.

12. Chang EF, Rieger JW, Johnson K, Berger MS, Barbaro NM, Knight RT. Categorical speech representation in human superior temporal gyrus. Nature Neuroscience. 2010;13(11):1428-U169.

13. Formisano E, De Martino F, Bonte M, Goebel R. "Who" is saying "what"? Brain-based decoding of human voice and speech. Science. 2008;322(5903):970-3.

14. Erez Y, Duncan J. Discrimination of Visual Categories Based on Behavioral Relevance in Widespread Regions of Frontoparietal Cortex. J Neurosci. 2015;35(36):12383-93.

15. Correia JM, Jansma BMB, Bonte M. Decoding Articulatory Features from fMRI Responses in Dorsal Speech Regions. J Neurosci. 2015;35(44):15015-25.
